# Gut microbiota dysbiosis exacerbates acute pancreatitis via *Escherichia coli*-driven neutrophil heterogeneity and NETosis

**DOI:** 10.1080/19490976.2025.2606480

**Published:** 2025-12-24

**Authors:** Yaoyu Zou, Nianshuang Li, Xueyang Li, Maobin Kuang, Xin Xu, Langyi Guan, Xin Li, Pan Zheng, Leyan Li, Jianhua Wan, Nonghua Lu, Jianping Liu, Cong He, Yin Zhu

**Affiliations:** a Department of Gastroenterology, Jiangxi Provincial Key Laboratory of Digestive Diseases, Jiangxi Clinical Research Center for Gastroenterology, Digestive Disease Hospital, The First Affiliated Hospital, Jiangxi Medical College, Nanchang University, Nanchang, Jiangxi, Peopel's Republic of China; b Postdoctoral Innovation Practice Base, The First Affiliated Hospital, Jiangxi Medical College, Nanchang University, Nanchang, Jiangxi, People's Republic of China

**Keywords:** Acute pancreatitis, Gut microbiota dysbiosis, *Escherichia coli*, Neutrophil extracellular traps, Neutrophil heterogeneity

## Abstract

Gut microbiota dysbiosis contributes to acute pancreatitis (AP) severity, but the specific microbes and mechanisms remain unclear. In this study, we employed both germ-free (GF) and specific-pathogen-free (SPF) murine models of AP to investigate the role of the intestinal microbiota. Our findings demonstrate that GF mice exhibited markedly attenuated pancreatic injury, inflammatory cell infiltration, and neutrophil extracellular traps (NETs) formation. Through fecal microbiota transplantation (FMT) from AP patients, differential antibiotic modulation, and single-bacterial colonization experiments, we identified Gram-negative bacteria, particularly *Escherichia coli (E. coli)*, as critical microbial drivers of disease exacerbation. Single-cell RNA sequencing revealed that microbiota dysbiosis profoundly reprogrammed both local pancreatic and systemic immune landscapes. Specifically, dysbiosis promoted emergency granulopoiesis in the bone marrow, enhanced neutrophil mobilization and activation, and facilitated the expansion of pro-inflammatory neutrophil subpopulations (Neutrophils_2 and Neutrophils_3). These subsets exhibited upregulated signaling through NETosis-associated pathways, including TLR, NF-κB, and IL-17 axes. Conversely, in GF conditions, we observed a predominance of an anti-inflammatory neutrophil subset (Neutrophils_4), characterized by the expression of tissue repair-associated genes such as *Reg1* and *Reg2*. Shotgun metagenomic profiling of fecal samples from patients with AP revealed an enrichment of *E. coli* during the acute phase, positively correlating with circulating cell-free DNA, a marker of NETosis. Together, these insights suggest that gut microbiota dysbiosis, notably increased *E. coli* abundance, may aggravate AP by reshaping immunity and promoting aberrant NETs formation, supporting microbiota or NETs targeted therapies.

## GRAPHICAL ABSTRACT

Summary diagram of gut microbiota dysbiosis–driven E. coli expansion reprograms systemic and pancreatic immunity to promote aberrant NETosis and aggravate acute pancreatitis.

**Figure:**
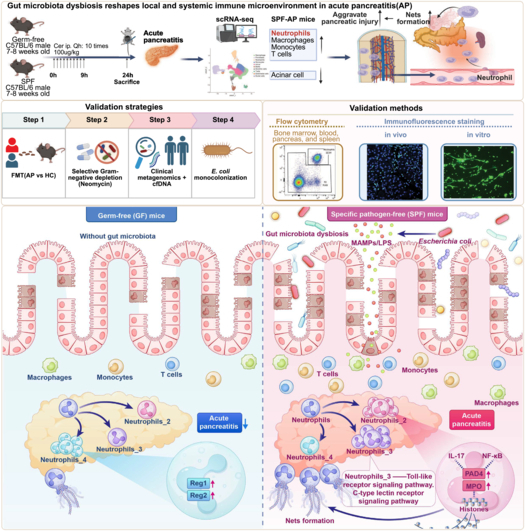

## Introduction

Acute pancreatitis (AP) is a frequently encountered gastrointestinal emergency, with a global incidence that has demonstrated a consistent upward trend over recent decades.^1‐3^ Current epidemiological estimates suggest an annual incidence ranging from 34 to 72 cases per 100,000 population annually,^4^ accompanied by a mortality rate of approximately 1.6 per 100,000 population^5^. While the majority of patients present with a mild, self-limiting form of the disease and recover without significant complications, an estimated 20% of cases advance to severe acute pancreatitis (SAP). This more critical form is characterized by extensive pancreatic necrosis, systemic inflammatory response syndrome (SIRS), and a heightened risk of multiple organ dysfunction syndrome (MODS), contributing significantly to a mortality rate of 30%–40%.^3^,^6^ Despite advances in supportive care, effective targeted therapeutic strategies remain lacking, highlighting an urgent need for the development of precision-based interventions to improve clinical outcomes in SAP.

Gastrointestinal dysfunction is a prevalent and clinically significant complication in patients with SAP, and is strongly associated with poor outcomes and increased mortality rates.^7^,^8^ The gut microbiota constitutes a fundamental component of the intestinal barrier, playing a pivotal role in the maintenance of mucosal homeostasis.^9^ Our previous study was among the first to document pronounced gut microbiota dysbiosis during the early stages of AP, characterized by a marked reduction in microbial diversity and a relative enrichment of opportunistic pathogens such as *Escherichia shigella, Enterococcus and Klebsiella*.^10^ Subsequent investigations have corroborated these findings, consistently demonstrating similar microbial alterations, and further implicating the expansion of specific pathogenic taxa in the progression and severity of AP.^11^,^12^

This pathogenic shift has been shown to promote regulatory T-cell (Treg) differentiation, thereby disrupting the Treg/Th17 balance, impairing intestinal barrier integrity, and facilitating the development of infectious pancreatic necrosis (IPN).^13^ Moreover, fecal microbiota transplantation (FMT) studies utilizing germ-free (GF) or antibiotic-pretreated murine models have provided causal evidence that microbiota dysbiosis exacerbates the severity of AP, underscoring its critical role in disease progression and the transition to severe forms of pancreatitis.^14^

Neutrophil extracellular traps (NETs) are filamentous, web-like structures composed of decondensed chromatin interlaced with neutrophil elastase, myeloperoxidase (MPO), and citrullinated histones. These structures are rapidly released by activated neutrophils in response to various stimuli, including pathogens, damage-associated molecular patterns (DAMPs), and platelet-derived signals. The formation of NETs is primarily mediated through a reactive oxygen species (ROS)-dependent signaling cascade involving MPO, neutrophil elastase (NE), and peptidylarginine deiminase 4 (PAD4).^15^ Aberrant activation of innate immune cells—particularly neutrophils and the formation of NETs— has been increasingly recognized as a key pathological mechanism contributing to the progression and severity of SAP.^16^ Excessive NETs formation not only exacerbates local pancreatic injury but also amplifies systemic inflammatory responses, thereby intensifying disease severity and promoting multi-organ dysfunction.^17^,^18^ Emerging evidence has demonstrated that transplantation of gut microbiota derived from patients with hypertriglyceridemia-induced pancreatitis into murine models significantly augments neutrophil infiltration and NETs formation. These findings suggest that specific gut microbial communities or their metabolites may modulate NETs activation and thereby influence SAP pathogenesis.^19^ Nevertheless, the precise microbial taxa responsible for initiating NETs formation and driving the progression to SAP remain incompletely characterized and warrant further investigation.

In this study, we investigated the role of gut microbiota dysbiosis, particularly the enrichment of *E. coli*, in exacerbating pancreatic injury and systemic inflammation during AP, focusing on neutrophil activation and NETs formation. Using germ-free mouse models, patient-derived fecal microbiota transplantation, selective antibiotic interventions, monocolonization assays, clinical metagenomic analysis, single-cell transcriptomics, and flow cytometry, we characterized immune responses associated with microbiota alterations. Our findings suggest that gut microbiota dysbiosis characterized by *E. coli* enrichment may contribute significantly to the severity of AP by promoting NETs-mediated inflammatory responses.

## Materials and methods

### Animals and experimental model of AP

All animal procedures were conducted in accordance with institutional ethical guidelines and approved by the Animal Ethics Committee of the First Affiliated Hospital of Nanchang University (Approval No. CDYFY-IACUC-202302QR052). Male C57BL/6 mice (7–8 weeks old), including both GF and specific pathogen-free (SPF) mice, were obtained from GemPharmatech Co., Ltd. (Nanjing, Jiangsu, China). SPF mice were housed in standard SPF animal facilities, while GF mice were maintained in sterile isolators under strictly controlled environmental conditions (temperature: 23 ± 2 °C; humidity: 40%–60%; light/dark cycle: 12 h/12 h), with *ad libitum* access to autoclaved food and water. Prior to experimentation, all mice were acclimated for one week. GF sterility was verified monthly, with additional checks before the start and at the end of each experiment. Fresh fecal samples and isolator swabs were examined by Gram staining and aerobic/anaerobic enrichment culture to confirm germ-free status in accordance with gnotobiotic standards.

AP was induced via hourly intraperitoneal injections of cerulein (Sigma-Aldrich; 100 μg/kg), administered over a period of 10 hours. Mice were euthanized 24 hours following the initial cerulein injection, and tissue and serum samples were collected for downstream analyzes.

### Human subjects

This study was conducted in accordance with the ethical standards of the institutional and national research committees and adhered to the principles outlined in the Declaration of Helsinki and the Belmont Report. Ethical approval was obtained from the Ethics Committee of the First Affiliated Hospital of Nanchang University (Approval Nos. 2020030 and 2023378), and written informed consent was obtained from all participants prior to enrollment.

Patients were eligible for inclusion if they were aged 18–70 years, presented within 72 hours of symptom onset, and were diagnosed with SAP based on the revised Atlanta criteria (2012),^20^ with a predicted severity defined by an APACHE II ≥ 8. Exclusion criteria included treatment with antibiotics within the preceding 14 days, pancreatitis secondary to endoscopic retrograde cholangiopancreatography (ERCP), pregnancy or lactation, a history of chronic pancreatitis, malignancy, or pre-existing severe cardiovascular, respiratory, renal, hepatic, or intestinal diseases. Patients with gastrointestinal bleeding, intestinal obstruction, or multiple organ failure (MOF) deemed imminently fatal by the attending physician were also excluded.

A total of 26 SAP patients and 26 healthy control subjects, matched by baseline clinical characteristics, were enrolled (**Table S1**). Stool and blood samples were collected from SAP patients at two time points: upon hospital admission (day 1, acute phase) and on day 14 (recovery phase). Samples from healthy controls were collected at a single time point. All samples were aliquoted and stored at −80 °C until subsequent analyzes.

### FMT in GF mice

Fresh fecal samples were obtained from five patients with SAP and five age- and sex-matched healthy volunteers (**Table S2**). In accordance with established protocols,^21^ equal amounts of fecal material from individuals within each group were pooled. One gram of pooled feces was homogenized in 5 mL of sterile phosphate-buffered saline (PBS) supplemented with 10% glycerol. The resulting suspension was filtered through a 70 μm cell strainer to remove particulate matter, then aliquoted and stored at −80 °C until use.

For FMT, GF mice were randomly assigned to receive either SAP- or healthy donor-derived microbiota. Each mouse was administered 200 μL of the corresponding fecal suspension via oral gavage once daily for 14 consecutive days. Six GF mice were included in each group. All procedures were performed under sterile conditions to maintain GF status.

### Differential antibiotic modulation of the gut microbiota

We performed antibiotic modulation of the mouse gut microbiota according to established protocols.^22^ Two antibiotic regimens were used: vancomycin (VAN, 0.5 g/L, Sigma-Aldrich; CAS 1404-93-9), which is predominantly active against Gram-positive (G^+^) bacteria, and neomycin (NEO, 1 g/L, Sigma-Aldrich; CAS 1405-10-3), which is mainly active against aerobic Gram-negative (G^−^) bacteria. Mice received daily antibiotic treatment via oral gavage (200 μL per mouse) for seven consecutive days, followed by induction of AP using cerulein. To confirm antibiotic-induced microbiota modulation, fecal samples were collected, and species-specific qRT-PCR was performed to quantify representative bacterial taxa, including *E. coli* and *Klebsiella pneumoniae*.

### Monocolonization with *Escherichia coli*

To evaluate the pathogenic role of *E. coli* in AP, GF mice were monocolonized with the clinically isolated *E. coli* strain ATCC 43895. Mice were administered 1 × 10^9^ CFU per day via oral gavage for seven consecutive days, a regimen based on established practices for stable intestinal colonization.^23^,^24^ Following colonization, the cerulein-induced AP model was established as previously described.

### Histopathological and immunohistochemical analyzes

Histological assessments were conducted following established protocols.^25^ Briefly, fresh pancreatic tissues were fixed in 4% paraformaldehyde for 24 hours, embedded in paraffin, sectioned, and subjected to hematoxylin and eosin (H&E) staining and immunohistochemical analyzes. Immunohistochemistry was performed using antibodies against Ly6G (1:250, Cell Signaling Technology, #87048S), myeloperoxidase (MPO, Abcam, ab9535), and F4/80 (Servicebio, GB11027-100). Histopathological scoring was based on the degree of edema, inflammatory cell infiltration, and necrosis. Immunohistochemical results were evaluated by combining staining intensity (0 = negative; 1 = weakly positive; 2 = moderately positive; 3 = strongly positive) with the proportion of positively stained areas (0 = 0%; 1 = 1–25%; 2 = 26–50%; 3 = 51–75%; 4 = 76–100%). The final immunohistochemistry score (range: 0-12) was calculated by multiplying the intensity and area scores. All assessments were independently performed by two trained pathologists blinded to group allocations. Representative images were acquired using a Nikon Eclipse microscope.

### Immunofluorescence staining

Paraffin-embedded pancreatic tissue sections (4 μm thick) were deparaffinized in xylene, rehydrated through an ethanol gradient, and incubated with 3% hydrogen peroxide at room temperature for 8 min to quench endogenous peroxidase activity. Antigen retrieval was subsequently achieved by heating in citrate buffer (pH 6.0) in a microwave for 15 min. Sections were permeabilized with 0.3% Triton X-100 at 37 °C for 15 min and blocked with 3% bovine serum albumin (BSA) at room temperature for 60 min. Sections were incubated overnight at 4 °C with primary antibodies against CitH3 (1:100, Abcam, ab5103), MPO (1:500, R&D, AF3667), Ly6G (1:800, BioLegend, 127602), Ly6C (1:100, Santa Cruz, sc-271811), F4/80 (Servicebio, GB11027-100), Amylase (1:100, Santa Cruz, sc-46657), and Reg3b (1:100, R&D, AF5110). After washing, sections were incubated with Alexa Fluor-conjugated secondary antibodies (Invitrogen) for 30 min at 37 °C. Finally, nuclei were counterstained with DAPI (Invitrogen). Images were captured using a LEICA Stellaris 5 confocal microscope.

### TUNEL assay

Apoptotic cells in pancreatic tissue sections were detected using a fluorescein-based TUNEL apoptosis detection kit (Servicebio, China), following the manufacturer's instructions. TUNEL-positive cells were visualized and quantified using a LEICA Stellaris 5 confocal microscope.

### Flow Cytometry

Single-cell suspensions were prepared from murine spleen, peripheral blood, and bone marrow, followed by filtering through a 70 μm cell strainer and erythrocyte lysis using red blood cell lysis buffer (Absin, abs9241-100 ml). Cells (~1 × 10⁷ per sample) were blocked with Fc receptor blocker (BD, Cat. No. 553141) and stained with the following fluorescently conjugated antibodies: viability dye BV510 (BD, 564406), CD45-APC-Cy7 (BD, 557659), CD3-FITC (BD, 553061), CD4-BV421 (BD, 562891), CD8-PerCP-Cy5.5 (BD, 551162), Foxp3-Alexa Fluor 647 (BD, 560401), CD25-PE (BD, 553075), CD11b-APC (BioLegend, 101212) and CD11b-PE-Cy7 (BioLegend, 101215), F4/80-PE (BD, 565410), and Ly6G-PerCP-Cy5.5 (BD, 560602). Intracellular staining for Foxp3 was performed using Foxp3 transcription factor fixation/permeabilization reagent (BD, 562574). Samples were analyzed using a NovoCyte D3000 flow cytometer (Agilent), and data were processed with FlowJo software (version 10.8.1). Gating strategies are detailed in **Figure S1**.

### Neutrophil isolation and NETs induction

Neutrophils were isolated from bone marrow using the mouse neutrophil isolation kit (STEMCELL, #19762). The purified neutrophils (1 × 10⁶ cells/well) were seeded into 24-well plates, stimulated with 100 nM PMA (Sigma-Aldrich) for 3 hours, and subsequently stained with 200 nM SYTOX Green dye. NETs formation was visualized via fluorescence microscopy.

### Serum biochemistry and inflammatory cytokine measurements

Orbital venous blood was collected and serum was separated for biochemical analyzes. Levels of serum amylase (C016-1) and lipase (A054-2) were determined using kits from Jiancheng Biotech, China. Serum cytokines, including IL-1β (E-EL-M0037c, Elabscience, China), IL-6 (E-EL-M0044c, Elabscience, China), TNF-α (E-EL-M3063, Elabscience, China), Lipopolysaccharide(LPS) (CSB-E13066m, Cusabio, China), and D-lactate (AB83429, Abcam, UK), were quantified using commercial ELIZA kits according to manufacturers' instructions. Additionally, serum levels of cell-free DNA (cfDNA), a surrogate marker of NETs, were measured using the Quant-iT PicoGreen® dsDNA kit (Invitrogen, P11496).

### Quantitative real-time PCR (qRT-PCR)

Total RNA was extracted from pancreatic tissue using TRIzol reagent (TIANGEN Biotech, Beijing, China) and reverse-transcribed into cDNA using a reverse transcription kit according to the manufacturer’s instructions. qRT-PCR was performed using a QuantStudio 5 real-time PCR system (Life Technologies), with GAPDH serving as an internal control. Primer sequences are listed in **Table S3**.

### Western blot analysis

Protein lysates from pancreatic tissue were prepared using RIPA lysis buffer, and concentrations were determined via BCA assay (ThermoFisher Scientific, U.S.A). Equal protein amounts were resolved by SDS-PAGE and transferred onto nitrocellulose membranes. Membranes were probed overnight with primary antibodies against NLRP3 (CST, D4D8T), TLR2 (CST, E1J2W), ASC (CST, D2W8U), Reg3b (Sino Biological, 51153-R005), and GAPDH (Proteintech, 10494-1-AP), followed by horseradish peroxidase (HRP)-conjugated secondary antibodies. Detection was performed using enhanced chemiluminescence (ECL) substrates and visualized on a ChemiDoc imaging system (BioRad, U.S.A).

### Tissue dissociation and single-cell sequencing

Fresh pancreatic tissues from mouse were collected and immediately immersed in DMEM medium (Gibco) supplemented with 10% fetal bovine serum (FBS), minced into approximately 0.5 mm³ fragments and enzymatically digested at 37 °C for 60 min in DMEM containing trypsin and collagenase I/II/IV (Gibco). The resulting cell suspensions were filtered through a 100 μm cell strainer (Corning) and centrifuged at 300 g for 5 min. Red blood cells were lysed by incubation with RBC lysis buffer (MACS) at 4 °C for 10 min, followed by another centrifugation step. Viable cells were quantified with a Luna cell counter, adjusted to 700–1200 cells/μl and used for library preparation with the Chromium Next GEM Single Cell 3ʹ Reagent Kits v3.1 (10 × Genomics, Cat#1000268). Libraries were subsequently sequenced sequenced using the Illumina NovaSeq 6000 platform (PE150).

### Single-cell RNA-seq data processing

Raw sequencing data were processed using Cell Ranger (10× Genomics) for barcode demultiplexing and constructing gene expression matrices. Downstream analyzes were conducted using the Seurat R package, with quality control filters excluding cells with <200 genes, <1000 UMIs, log_10_ (Genes/UMI) <0.7, >20% mitochondrial or >5% hemoglobin gene content. Potential doublets were identified and removed using DoubletFinder (v2.0.2), resulting in high-quality cells for subsequent analyzes (**Table S4**). Highly variable genes were determined by Seurat, and used for principal component analysis (PCA) and Uniform Manifold Approximation and Projection (UMAP) dimensionality reduction. Cell clusters were defined, and cluster-specific marker genes were identified using the FindAllMarkers function, with visualizations performed using the VlnPlot and FeaturePlot functions. Cell type annotation was conducted using SingleR package based on publicly available reference datasets.

### Pathway enrichment analyzes

To reveal functional differences between distinct cell subsets, Gene Ontology (GO), Kyoto Encyclopedia of Genes and Genomes (KEGG), and Gene Set Enrichment Analysis (GSEA) were applied. GO analyzes assessed biological processes, cellular components, and molecular functions. KEGG pathway analysis identified enriched signaling pathways, and GSEA was used to evaluate enrichment of predefined gene sets among differentially expressed genes. In addition, we used the NETosis gene set derived from the KEGG pathway map04613 (Neutrophil extracellular trap formation) to characterize NETosis-related transcriptional activity.

### Metagenomic sequencing and analysis

Bacterial genomic DNA was extracted from fecal samples using the OMEGA Mag-Bind Soil DNA Kit (Omega Bio-Tek, Norcross, GA, U.S.A) following the manufacturer’s instructions. Metagenomic sequencing was then performed on the MGISEQ-T7 platform (OE Biotech Co., Ltd., Shanghai, China). Raw sequencing data underwent quality control steps including removal of adapter sequences, low-quality reads, and host genomic contamination. The sequencing data volume ranged between 8.13 and 18.91 Gb per sample, with Q20 and Q30 scores of 96.47% and 89.91%, respectively. And the contig N50 length ranged from 291 to 23,304 bp. Non-redundant gene sets (n = 4,033,045) were constructed after redundancy removal, with an average gene length of 628.13 bp. Taxonomic annotations were performed against the NCBI NR database.

### Statistical analysis

All data were expressed as mean ± standard error of the mean (SEM). Between-group comparisons were assessed using Student’s t-test (for normally distributed data) or Mann–Whitney U test (for non-normally distributed data), as appropriate. For multiple comparisons, p-values were adjusted using the false discovery rate method. Correlations between microbial abundance and clinical parameters were analyzed using Spearman’s correlation. Statistical analyzes were performed using GraphPad Prism 8.0 and R software (version 3.6.1), with a two-sided p-value <0.05 considered statistically significant.

## Results

### GF mice exhibited attenuated pancreatic injury and inflammation versus SPF control in experimental AP model

**Figure 1.**
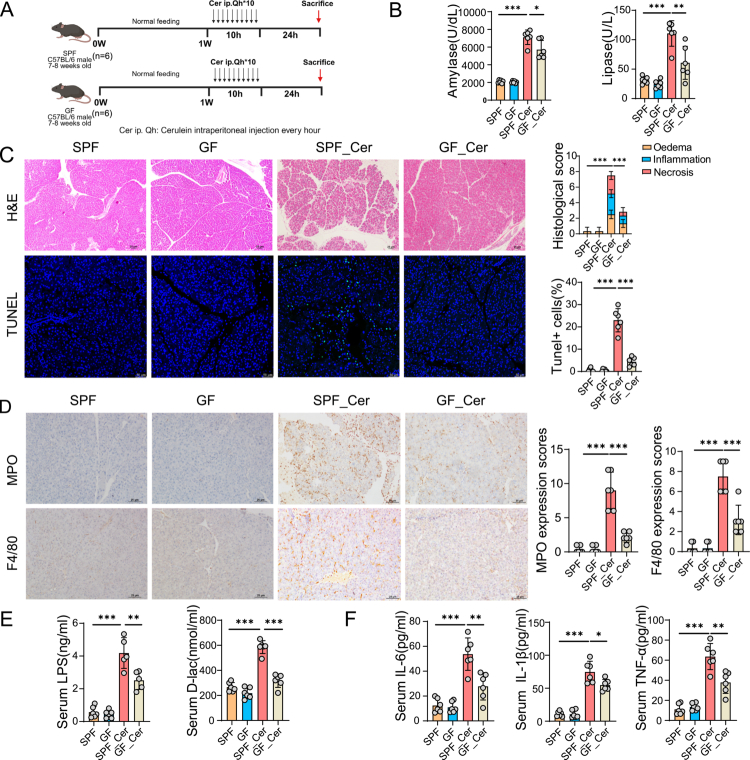
GF conditions attenuate Cer-induced AP and systemic inflammation. (A) Schematic overview of the experimental design. Male C57BL/6 mice (7–8 weeks old) housed under SPF or GF conditions were administered hourly intraperitoneal injections of cerulein (100 μg/kg) for 10 hours. Mice were euthanized 24 hours after the final injection for subsequent analyzes (n = 6). (B) Serum levels of pancreatic enzymes (amylase and lipase) were significantly lower in GF_Cer mice than in SPF_Cer mice, indicating attenuated pancreatic injury. (C) Representative H&E staining of pancreatic tissue (scale bar: H&E, 25 μm; TUNEL, 50 μm) revealed histopathological differences between groups, with semi-quantitative scoring of edema, inflammatory infiltration, and acinar necrosis. Apoptosis was assessed by TUNEL staining with quantification. (D) Immunohistochemical analyzes of pancreatic tissue revealed reduced infiltration of MPO⁺ neutrophils and F4/80⁺ macrophages (scale bars: 25 μm). (E) Markers of intestinal barrier dysfunction, including serum LPS and D-lactate, were also significantly reduced under GF conditions. (F) Circulating levels of pro-inflammatory cytokines (IL-6, IL-1β, and TNF-α) were markedly decreased in GF mice, reflecting suppression of systemic inflammatory responses. Data are presented as mean ± SEM. Statistical comparisons were made using an unpaired two-tailed Student’s t-test. *p < 0.05, **p < 0.01, ***p < 0.001. Abbreviations: GF, germ-free; SPF, specific pathogen-free; Cer, cerulein; AP, acute pancreatitis; H&E, hematoxylin and eosin; MPO, myeloperoxidase; LPS, lipopolysaccharide; IL, interleukin; TNF-α, tumor necrosis factor-alpha.

Emerging evidence suggests that gut microbiota dysbiosis plays a critical role in the pathogenesis of AP in both murine models and human patients.^10^ To elucidate the contribution of gut microbiota dysbiosis to AP progression, we employed a cerulein-induced AP models in GF and SPF male C57BL/6 mice (Figure 1A). Compared with SPF-Cer mice (SPF mice with cerulein-induced AP), GF-Cer mice (GF mice with cerulein-induced AP) exhibited significantly reduced serum amylase and lipase levels, indicative of attenuated pancreatic injury (Figure 1B). Consistent with these biochemical results, histopathological analyzes demonstrated reduced pancreatic edema, inflammatory infiltration, and acinar cell necrosis in GF-Cer mice, accompanied by decreased acinar cell apoptosis as revealed by TUNEL staining (Figure 1C). Furthermore, GF-Cer exhibited markedly reduced expression of MPO and F4/80 compared to SPF controls (Figure 1D). Intestinal barrier injury markers LPS and D-lactate, were also lower in GF-Cer mice vs SPF-Cer controls (Figure 1E). Correspondingly, GF mice showed consistently decreased serum levels of inflammatory cytokines IL-6, IL1β and TNF-α (Figure 1F). Collectively, these data demonstrate that GF mice exhibited attenuated pancreatic injury and inflammation versus SPF controls in the experimental AP model.

### ScRNA-seq profiling reveals gut microbiota dysbiosis reprograms immune microenvironment in murine AP

**Figure 2.**
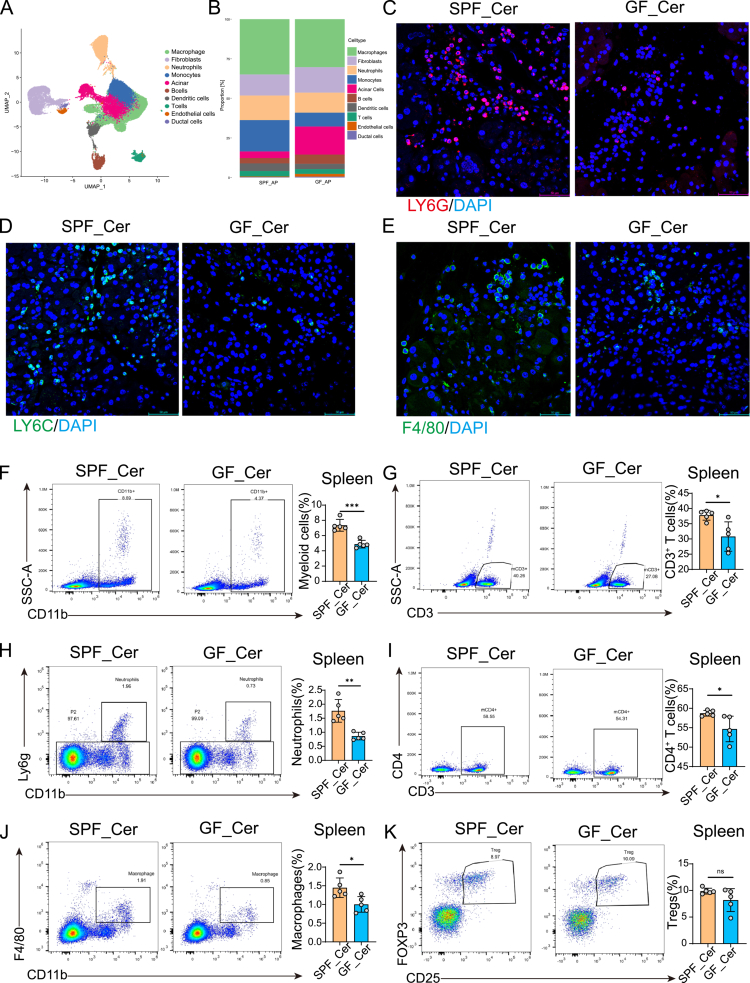
scRNA-seq profiling reveals gut microbiota dysbiosis reprograms the immune microenvironment in murine AP. (A) UMAP visualization of pancreatic single-cell RNA sequencing, identifying major cell populations including neutrophils, macrophages, monocytes, fibroblasts, acinar cells, ductal cells, endothelial cells, dendritic cells, T cells, and B cells. (B) Comparative cellular composition analysis between SPF_AP and GF_AP groups demonstrated a marked reduction in myeloid and inflammatory cell subsets in GF_AP mice, indicating microbiota-dependent immune activation. (C–E) Representative immunofluorescence staining of pancreatic tissues for neutrophils (Ly6G), monocytes (Ly6C), and macrophages (F4/80), confirming reduced infiltration of inflammatory cells under GF conditions (scale bar, 50 μm). (F–K) Flow cytometric analysis of splenic immune populations: CD11b⁺ myeloid cells (F), CD3⁺ T cells (G), CD11b⁺Ly6G⁺ neutrophils (H), CD4⁺ helper T cells (I), CD11b⁺F4/80⁺ macrophages (J), and CD4⁺CD25⁺Foxp3⁺ regulatory T cells (K). Bar plots depict the relative frequency of each subset among CD45⁺ immune cells. Data are presented as mean ± SEM. Statistical significance was determined using unpaired two-tailed Student’s t-test. *p < 0.05, **p < 0.01, ***p < 0.001. Abbreviations: GF, germ-free; SPF, specific pathogen-free; AP, acute pancreatitis; UMAP, Uniform Manifold Approximation and Projection; scRNA-seq, single-cell RNA sequencing.

Accumulating evidence indicates that the gut microbiota can influence pathological processes in distant organs through immune-mediated mechanisms.^26^,^27^ To directly compare cellular heterogeneity and molecular phenotypes in pancreatic tissues of AP mice under GF versus SPF conditions, scRNA-seq on pancreatic tissues from both cohorts was conducted. A total of five pancreatic tissues from mice with cerulein-induced AP (GF, n = 3; SPF, n = 2) were enrolled in this study. Following quality control and filtering, a total of 52,364 cells were retained for downstream analysis (median, 11,418 cells per mouse; range, 5,243–15,364) **(Table S4)**. Unsupervised clustering with UMAP dimensionally reduction resolved 16 distinct cell clusters, corresponding to the expected pancreatic cell types, including macrophages (*C1qa, C1qb, C1qc*), fibroblasts (*Col1a1, Col1a2, Lum*), neutrophils (*S100a8, G0s2, Cxcr2*), monocytes (*Ly6c2, Ccr2, S100a4*), pancreatic acinar cells (*Prss2, Celea3b, Cpa1*), B cells (*Cd79a, Cd79b, Ms4a1*), dendritic cells (*Runx2, Cd74, H2-Aa*), T cells (*Cd3e, Cd3g, Cd3d*), endothelial cells (*Pecam1, Plvap, Cdh5*), and ductal cells (*Krt19, Sox9, Ambp*) (Figure 2A and S2A). To better understand pancreatic microenvironment landscape, we defined the abundance and composition of major cell lineages across experimental groups. Notably, there was a significant reduction in immune populations, including neutrophils, macrophages, monocytes, and T cells, concomitant with increased proportion of acinar cells in GF-Cer mice relative to SPF-Cer controls (Figure 2B). Supporting our transcriptome analysis, immunofluorescence staining confirmed reduced infiltration of neutrophils, monocytes and macrophage, as evidence by decreased expression of LY6G、LY6C and F4/80, respectively (Figure 2C-E) in GF mice with AP. Marker gene analysis confirmed the distinct expression profiles of each cellular subpopulation (Figure S2B).

**Figure 3.**
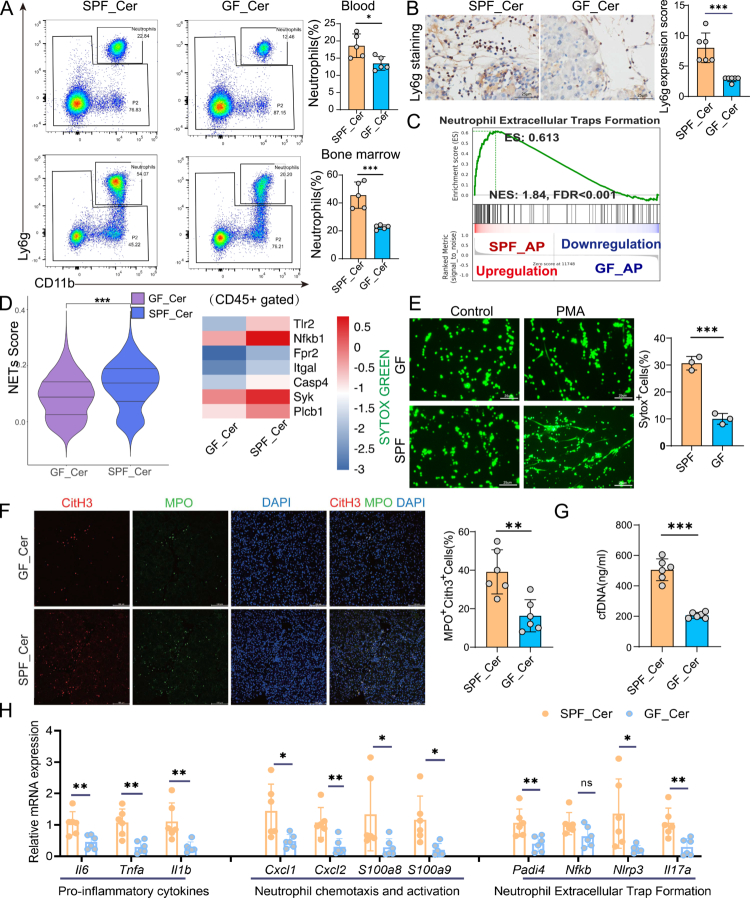
Gut microbiota dysbiosis enhances neutrophil recruitment and NETs formation in murine AP. (A) Flow cytometric analysis and quantification of neutrophil (CD11b+Ly6g^+^) in peripheral blood and bone marrow from SPF_Cer and GF_Cer mice, demonstrating increased neutrophil mobilization under dysbiotic conditions. (B) Representative immunohistochemical staining of Ly6g-positive neutrophil infiltration in pancreatic tissue, with corresponding quantification, indicating elevated neutrophil infiltration in SPF_Cer mice (scale bar, 20 μm). (C) GSEA illustrating significant enrichment of NETS formation-related pathways in SPF_AP mice compared to GF_AP mice. (D) Violin plot comparing NETs-related gene signature scores, alongside a heatmap illustrating differential expression of key NETs-associated genes including *Tlr2, Nfkb1, Fpr2, Itgal, Casp4, Syk, Plcb1*, between SPF_Cer and GF_Cer mice. (E) SYTOX Green staining and quantification of bone marrow-derived neutrophils following PMA stimulation, revealing enhanced NETs release in SPF mice (scale bar, 50 μm). (F) Immunofluorescence staining and quantification of NETs formation (CitH3^+^MPO^+^ cells) in pancreatic tissues, confirming increased *in situ* NETs formation in SPF_Cer mice (scale bar, 50 μm). (G) Serum levels of cfDNA, a systemic NET biomarker, measured by ELISA, were significantly elevated in SPF_Cer mice. (H) qRT-PCR analysis of pancreatic tissues demonstrating increased expression of pro-inflammatory cytokines (*Il6, Tnfa* and *Il1β*), neutrophil chemotaxis and activation markers (*Cxcl1, Cxcl2, S100a8* and *S100a9*), and NETosis-associated genes (*Padi4, Nfkb, Nlrp3* and *Il17a*) in SPF_Cer mice. Data are presented as mean ± SEM. Statistical significance was determined using unpaired two-tailed Student’s t-test; *p <0.05, **p<0.01, ***p<0.001. Abbreviations: SPF, specific pathogen-free; GF, germ-free; AP, acute pancreatitis; NETs, neutrophil extracellular traps; GSEA, gene set enrichment analysis; PMA, phorbol 12-myristate 13-acetate; CitH3, citrullinated histone H3; MPO, myeloperoxidase; cfDNA, cell-free DNA; qRT-PCR, real-time quantitative PCR.

To avoid cell-level pseudo-replication, we aggregated single-cell transcript counts at the sample (mouse) level to create a pseudo-bulk expression matrix and performed differential-expression analysis. KEGG enrichment analyzes revealed that genes upregulated in SPF-Cer tissues were predominantly associated with inflammatory and innate immune signaling pathways, including TNF, NF-κB, chemokine signaling, IL-17, Toll-like receptors, and NOD-like receptors (**Figure S2C-D**). GO analyzes similarly demonstrated enrichment in immune-related biological processes such as chemokine-mediated cell migration and inflammatory responses (**Figure S2E-F**). Heatmap visualization of the top 20 differentially expressed genes further indicated elevated expression of proinflammatory mediators— such as *Nlrp3* and *Cxcl1*—in SPF tissues, whereas tissue repair including Reg3b and Prss2 were more highly expressed in GF tissues (**Figure S3A**). Since transcriptional differences do not necessarily correspond directly to protein abundance, we next validated these gene signatures at the protein level. Western blot analysis confirmed markedly increased protein levels of TLR2, NLRP3, and ASC, alongside reduced expression of the epithelial regeneration factor Reg3β, in the pancreatic tissues of SPF mice (**Figure S3B**). We then leveraged single-cell resolution analyzes to identify specific cellular sources of these key proteins. UMAP plots and violin plots indicate that *Reg3b* expression is specifically enriched in pancreatic acinar cells, with higher transcript levels in GF-Cer than SPF-Cer tissues (**Figure S3C–D**). Immunofluorescence co-staining with amylase confirms Reg3β protein localization predominantly in acinar cells (**Figure S3E**). Conversely, *Nlrp3* expression is mainly detected in macrophages, neutrophils, dendritic cells, and monocytes, showing marked upregulation in SPF-Cer pancreata (Figure 3F–G), with immunofluorescence further validating increased NLRP3 protein in neutrophils (**Figure S3H**).

To assess the systemic immunological impact of gut microbiota dysbiosis, flow cytometric profiling was performed on splenocytes. SPF mice exhibited significantly higher frequencies of CD11b^+^ myeloid cells (Figure 2F), CD3^+^ T cells (Figure 2G), Ly6G^+^ neutrophils (Figure 2H), and F4/80^+^ macrophages (Figure 2J), compared to GF mice. A moderate increase in CD4^+^ T cells was also observed (Figure 2I), while the frequency of regulatory T cells (CD25^+^Foxp3^+^) showed no significant difference between groups (Figure 2K).

Collectively, these findings demonstrate that gut microbiota dysbiosis profoundly remodels both the local pancreatic and systemic immune landscapes in AP. The dysbiotic microbiota promotes immune activation, particularly of inflammatory myeloid populations, while concurrently impairing reparative responses within the pancreatic tissue microenvironment.

### Gut microbiota dysbiosis induces emergency granulopoiesis and promotes NETs formation in AP

To investigate the contribution of gut microbiota dysbiosis to neutrophilic responses in AP, we performed flow cytometry analysis of hematopoietic and peripheral immune compartments. Compared with GF counterparts, SPF mice exhibited significantly elevated frequencies of CD45^+^CD11b^+^Ly6G^+^ neutrophils in the bone marrow, peripheral blood, and spleen following AP induction, indicative of inflammation-driven emergency granulopoiesis, and enhanced systemic neutrophil mobilization (Figure 3A). Immunohistochemical staining for Ly6G further confirmed robust neutrophils infiltration into pancreatic lesions in SPF mice (Figure 3B).

Gene set enrichment analysis (GSEA) of pancreatic pseudo-bulk expression matrix revealed marked enrichment of NETosis-associated pathways in SPF mice (Figure 3C). Heatmap visualization of differentially expressed genes demonstrated upregulation of canonical NETosis-associated transcripts, accompanied by significantly elevated NETosis activation scores (Figure 3D). These findings were further validated by qRT–PCR analyzes of representative NETosis-associated genes in pancreatic tissues **(Figure S4A)**. Functionally, bone marrow-derived neutrophils from SPF mice exhibited significantly heightened NETotic responses upon stimulation with phorbol myristate acetate (PMA), as determined by SYTOX Green staining (Figure 3E).

We next performed immunofluorescence co-staining for MPO and CitH3, hallmarks of NETs structures, was notably increased in pancreatic sections from SPF mice (Figure 3F). In addition, circulating levels of cell-free DNA (cfDNA), a systemic biomarker of NETosis, were significantly elevated in SPF mice (Figure 3G). qRT-PCR further validated these findings, showing increased expression of pro-inflammatory cytokines (*Il6, Tnfa, Il1b*), chemokines (*Cxcl1, Cxcl2*), and genes implicated in neutrophil activation and NETs formation, including *S100a8, S100a9, Padi4, Nfkb, Nlrp3, Il17a* in pancreatic tissues from SPF mice (Figure 3H).

Collectively, these findings indicate that gut microbiota dysbiosis induces emergency granulopoiesis and robust neutrophil activation, thereby amplifying NETs formation. This excessive neutrophilic response contributes to the exacerbation of both local and systemic inflammation during AP progression.

### FMT confirms that dysbiotic gut microbiota from AP patients exacerbates pancreatic injury and NETs formation

**Figure 4.**
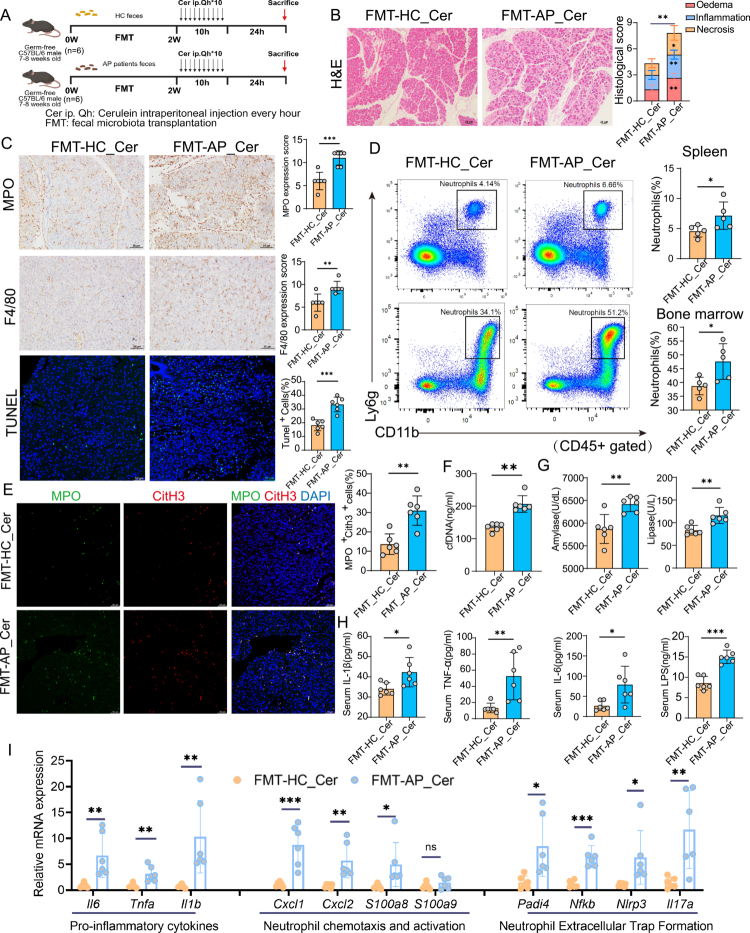
FMT from AP patients exacerbates pancreatic injury and NETs formation in GF mice. (A) Schematic representation of experimental design: GF mice received FMT from either healthy controls (FMT-HC) or acute pancreatitis (FMT-AP) patients for two weeks. AP was induced by 10 hourly intraperitoneal injections of Cer (100 μg/kg). Mice were sacrificed 24 hours after the initial injection. (B) Representative H&E staining of pancreatic tissues (scale bar, 25 μm), with histological scores of edema, inflammatory infiltration, and necrosis. (C) Immunohistochemical analysis and quantification of pancreatic infiltration by neutrophils (MPO), macrophages (F4/80), and apoptotic cell death (TUNEL assay) (scale bars: MPO, F4/80, 25 μm; TUNEL, 50 μm). (D) Flow cytometric analysis and quantification of CD11b^+^Ly6G^+^ neutrophils (gated on CD45^+^ cells) in spleen and bone marrow. (E) Immunofluorescence staining and quantification of NETs formation in pancreatic tissue, defined by co-expression of citrullinated histone H3 (CitH3) and MPO (CitH3^+^MPO^+^ cells; scale bar, 50 μm). (F) Serum cfDNA concentrations, serving as an indicator of systemic NETs release. (G) Serum levels of amylase and lipase, indicative of pancreatic injury severity. (H) Serum levels of pro-inflammatory cytokine (TNF-α, IL-6, IL-1β) and LPS, reflecting systemic inflammation and intestinal barrier disruption. (I) qRT-PCR analysis of pancreatic tissue for pro-inflammatory cytokines *(Il6, Tnfa, Il1β*), neutrophil chemotaxis and activation markers (*Cxcl1, Cxcl2, S100a8, S100a9*), and NETs-related genes (*Padi4, Nfkb, Nlrp3, Il17a*). Data are presented as mean ± SEM. Statistical significance was determined using a two-tailed unpaired Student’s t-test (*p<0.05, **p<0.01, ***p<0.001). Abbreviations: FMT, fecal microbiota transplantation; AP, acute pancreatitis; GF, germ-free; Cer, cerulein; HC, healthy control; MPO, myeloperoxidase; TUNEL, terminal deoxynucleotidyl transferase dUTP nick-end labeling; CitH3, citrullinated histone H3; cfDNA, cell-free DNA; LPS, lipopolysaccharide; NETs, neutrophil extracellular traps; qRT-PCR, real-time quantitative PCR.

To directly evaluate the pathological impact of gut microbiota dysbiosis observed in AP, we performed FMT experiments using GF mice. GF recipients were colonized with fecal microbiota from either healthy controls (FMT-HC) or patients with AP (FMT-AP), followed by cerulein-mediated AP induction (Figure 4A). Compared to FMT-HC controls, mice receiving FMT-AP exhibited significantly exacerbated pancreatic injury, characterized by increased interstitial edema, extensive inflammatory cell infiltration, and acinar necrosis, as reflected by elevated histopathological scores (Figure 4B).

Immunohistochemical analysis revealed enhanced infiltration of MPO^+^ neutrophils and F4/80^+^ macrophages, along with increased acinar cell apoptosis in the pancreatic tissues of FMT-AP mice (Figure 4C). Flow cytometric analysis further demonstrated significantly elevated proportions of CD11b^+^Ly6G^+^ neutrophil in both the spleen and bone marrow from FMT-AP mice, indicating heightened systemic neutrophil mobilization (Figure 4D). Markers of NETs formation—including MPO/CitH3 double-positive cells in pancreatic tissues and serum cfDNA levels—were also markedly increased in the FMT-AP group (Figure 4E–F).

Serum biochemical analysis revealed significantly elevated levels of amylase, lipase, inflammatory cytokines (IL-6, TNF-α, IL-1β), and gut barrier dysfunction marker (LPS) in FMT-AP mice relative to controls (Figure 4G–H). Moreover, qRT-PCR analyzes of pancreatic tissues confirmed significant upregulation of inflammatory cytokine genes (*Il6, Tnfa, Il1b*), chemokines (*Cxcl1, Cxcl2*), and key NET-associated genes (*Padi4, Nfkb, Nlrp3, Il17a*) in the FMT-AP group (Figure 4I). Taken together, these findings provide direct evidence that gut microbiota dysbiosis from AP patients exacerbates pancreatic injury and augments NETs formation, thereby intensifying both local pancreatic and systemic inflammatory responses.

### Gram-negative bacteria are key drivers of SAP

**Figure 5.**
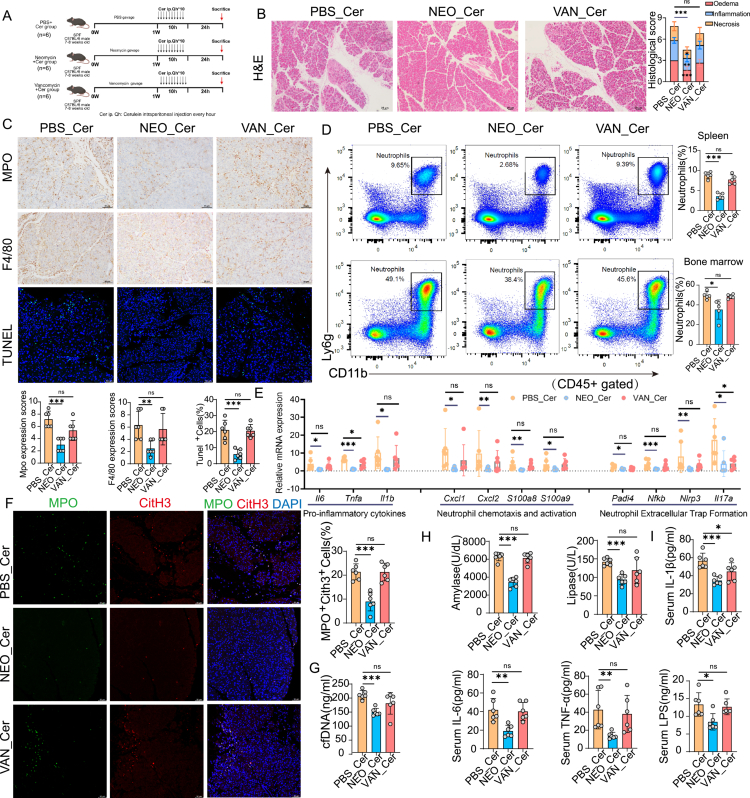
Selective depletion of Gram-negative bacteria attenuates cerulein-induced pancreatic inflammation and NETs formation, whereas depletion of Gram-positive bacteria fails to confer protection. (A) Schematic overview of the experimental design: SPF mice were pretreated orally with PBS, neomycin (NEO; targeting Gram-negative bacteria), or vancomycin (VAN; targeting Gram-positive bacteria) for one week, followed by hourly intraperitoneal injections of cerulein (Cer, 100 μg/kg, 10 doses). Mice were sacrificed 24 h after the initial injection. (B) Representative H&E staining of pancreatic tissue (scale bar, 25 μm) with corresponding histological scoring for edema, inflammatory infiltration, necrosis. (C) Immunohistochemical staining and quantification of pancreatic neutrophils (MPO), macrophages (F4/80), and apoptotic cells (TUNEL assay) (scale bars: MPO and F4/80, 25 μm; TUNEL, 50 μm). (D) Flow cytometric analysis and quantification of neutrophils (CD11b^+^Ly6G^+^, gated on CD45^+^ cells) in spleen and bone marrow. (E) qRT-PCR analysis of pancreatic expression levels of pro-inflammatory cytokines (*Il6, Tnfa, Il1β*), neutrophil chemotaxis and activation markers (*Cxcl1, Cxcl2, S100a8, S100a9*), and genes involved in NETosis (*Padi4, Nfkb, Nlrp3, Il17a*). (F) Immunofluorescence staining and quantification of NETs formation (MPO^+^CitH3^+^ cells) in pancreatic tissues (scale bar, 50 μm). (G) Serum concentrations of cfDNA assessed by ELISA. (H–I) Serum levels of pancreatic enzymes (amylase and lipase) and systemic inflammatory markers (TNF-α, IL-6, IL-1β), as well as lipopolysaccharide (LPS), indicating intestinal barrier disruption. Data are presented as mean ± SEM. Statistical significance was determined by an unpaired two-tailed Student’s t-test (*p<0.05, **p<0.01, ***p<0.001; ns, not significant). Abbreviations: AP, acute pancreatitis; SPF, specific pathogen-free; Cer, cerulein; NEO, neomycin; VAN, vancomycin; MPO, myeloperoxidase; TUNEL, terminal deoxynucleotidyl transferase dUTP nick-end labeling; CitH3, citrullinated histone H3; cfDNA, cell-free DNA; NETs, neutrophil extracellular trap; qRT-PCR, real-time quantitative PCR; LPS, lipopolysaccharide.

To delineate the contribution of distinct bacterial taxa to the progression of AP, we selectively depleted G^−^ and G^+^ bacteria using NEO and VAN, respectively, prior to cerulein-induced AP (Figure 5A). Species-specific fecal qRT-PCR confirmed the targeted depletion of gut bacteria (**Figure S4B**). Mice pre-treated with NEO exhibited significantly attenuated pancreatic injury, as evidenced by reduced interstitial edema, inflammatory infiltration, and acinar cell apoptosis compared to the PBS control group (Figure 5B–C). In contrast, VAN treatment had minimal impact on histopathological severity (Figure 5B–C). VAN treatment showed no significant improvement. Flow cytometric analysis further revealed that NEO administration significantly decreased the proportion of CD11b⁺Ly6G⁺ neutrophils in both spleen and bone marrow compartments (Figure 5D), indicating suppressed emergency granulopoiesis and neutrophil mobilization. At the molecular level, qRT-PCR analysis of pancreatic tissues showed that NEO treatment markedly downregulated the expression of pro-inflammatory cytokines (*Il6*, *Tnfa*, *Il1b*), neutrophil-recruiting chemokines (*Cxcl1, Cxcl2, S100a8* and *S100a9*), and key genes associated with NETs formation (*Padi4, Nfkb, Nlrp3* and *Il17a*) (Figure 5E). Consistently, immunofluorescence staining demonstrated a significant reduction in pancreatic NETs formation (MPO/CitH3 co-localization), accompanied by lower circulating levels of serum cfDNA in Neo-treated mice (Figure 5F–G). Moreover, serum biochemical indices—including amylase, lipase, and pro-inflammatory cytokines (TNF-α, IL-6 and Il1β), as well as the gut barrier dysfunction marker LPS—were significantly reduced following G^−^ bacterial depletion (Figure 5H). Together, these findings establish G^−^ bacteria as dominant microbial contributors to the pathogenesis of SAP, and highlight the therapeutic potential of selectively targeting G^−^ microbiota to mitigate pancreatic injury and systemic inflammation.

### Enrichment of *Escherichia coli* in acute-phase AP and its positive correlation with circulating cfDNA levels

**Figure 6.**
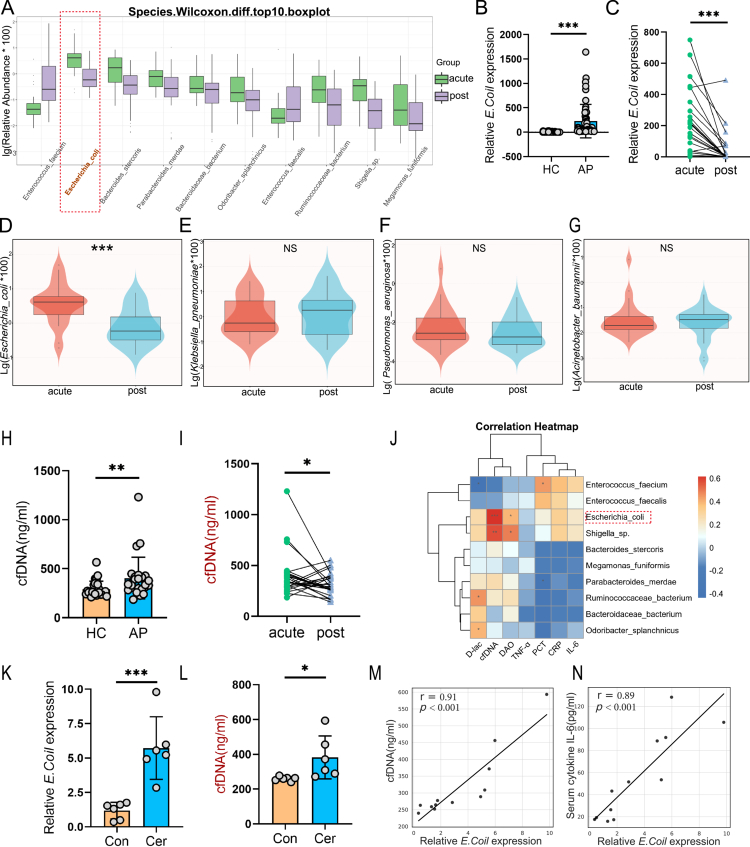
Enrichment of *Escherichia coli* during acute-phase AP correlates with increased serum cfDNA as a marker of NETs formation. (A) Metagenomic sequencing of fecal samples from AP patients revealed the top 10 bacterial species significantly altered between the acute phase (day of onset) and recovery phase (day 14), as determined by Wilcoxon rank-sum test. (B–C) qRT-PCR validation of intestinal *E. coli* abundance in healthy controls (HC) and AP patients (B), and in paired samples from AP patients during acute and recovery phases (C). (D–G) Violin plots comparing the relative abundance of common Gram-negative pathogens—including *E. coli, Klebsiella pneumoniae, Pseudomonas aeruginosa*, and *Acinetobacter baumannii*—between acute and recovery phases of AP. (H–I) Measurement of serum cfDNA in AP patients compared to healthy controls (H), and in paired acute versus recovery phase samples (I). (J) Correlation heatmap depicting the relationship between *E. coli* abundance and NETs- associated biomarkers (cfDNA and pro-inflammatory cytokines) in clinical samples. (K–L) Relative intestinal *E. coli* abundance and serum cfDNA levels in a murine cer-induced P model. (M–N) Correlation analyzes between intestinal *E. coli* levels and serum cfDNA (M) or IL-6 levels (N) in mice. Data are presented as mean ± SEM. Statistical significance was determined using an unpaired two-tailed Student’s t-test. *p<0.05, **p<0.01, ***p<0.001; NS, not significant. Abbreviations: AP, acute pancreatitis; cfDNA, cell-free DNA; NETs, neutrophil extracellular traps; qRT-PCR, real-time quantitative PCR; HC, healthy controls; Conn, control; Cer, cerulein; IL-6, interleukin-6; SEM, standard error of mean.

To delineate temporal alterations in gut microbiota composition during the clinical course of acute AP, we performed longitudinal metagenomic sequencing on fecal samples collected from patients at the acute phase (day of onset) and recovery phase (day 14). Among the top 10 species exhibiting significant differential abundance between these two phases (Wilcoxon rank-sum test), *E. coli* demonstrated the most pronounced dynamic shift—markedly enriched during the acute phase and significantly reduced during recovery (Figure 6A, red dashed box). Focused analysis of clinically relevant G^−^ bacterial species revealed that *E. coli* was the only pathogen significantly diminished during the recovery phase (Figure 6D), while *Klebsiella pneumoniae* (Figure 6E), *Pseudomonas aeruginosa* (Figure 6F), and *Acinetobacter baumannii* (Figure 6G) showed no statistically significant changes, suggesting a specific role of *E. coli* in AP progression. qRT-PCR further validated significantly elevated *E. coli* abundance in fecal samples from AP patients during the acute phase compared to healthy controls (Figure 6B), with a notable reduction observed by day 14 (Figure 6C). Concomitantly, levels of circulating cfDNA, a marker of NETs, were significantly increased in the acute phase (Figure 6H) and decreased during recovery (Figure 6H-I), displaying a robust positive correlation with intestinal *E. coli* abundance (Figure 6J). These findings were corroborated in murine AP models, where intestinal *E. coli* abundance and serum cfDNA levels were both significantly elevated following AP induction (Figure 6K–L). Furthermore, strong positive correlation were observed between *E. coli* abundance, serum cfDNA, and systemic levels of the inflammatory cytokine IL-6 (Figure 6M–N). Taken together, these results highlight intestinal *E. coli* as a dynamically regulated member of the gut microbiota in AP, whose enrichment during the acute phase is closely associated with heightened NETs formation and systemic inflammation, suggesting *E. coli* as a potential microbial driver of disease exacerbation of AP.

### *Escherichia coli* exacerbates cerulein-induced pancreatic injury and promotes NETs formation in AP

**Figure 7.**
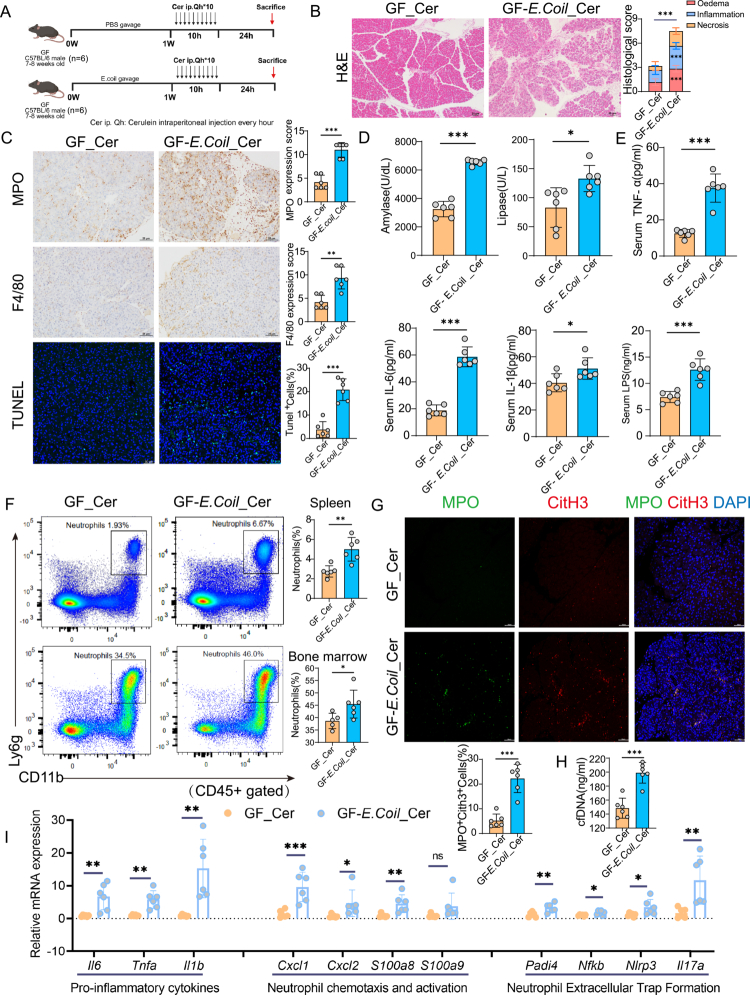
*E. coli* exacerbates cerulein-induced AP and promotes NETs formation in GF mice. (A) Schematic overview of the experimental protocol: GF mice were orally administered PBS or a pathogenic strain of *E. coli* (ATCC 43895) for one week, followed by hourly intraperitoneal injections of cerulein (100 μg/kg, 10 doses in total). Mice were sacrificed 24 h after the initial injection. (B) Representative H&E staining of pancreatic tissues (scale bar, 25 μm), with corresponding histological scoring for edema, inflammatory infiltration, and necrosis. (C) Immunohistochemical staining and quantification of neutrophils (MPO), macrophages (F4/80), and apoptotic cells (TUNEL assay) in pancreatic tissue sections (scale bars: MPO and F4/80, 25 μm; TUNEL, 50 μm). (D) Serum amylase and lipase levels as biochemical indicators of pancreatic injury. (E) Serum concentrations of inflammatory cytokines (TNF-α, IL-6, IL-1β) and LPS, reflecting systemic inflammatory responses and gut barrier disruption. (F) Flow cytometric analysis and quantification of CD11b^+^Ly6G^+^ neutrophils (gated on CD45^+^ cells) in spleen and bone marrow. (G) Immunofluorescence staining and quantification of pancreatic tissue showing NETs formation (CitH3^+^MPO^+^ cells) (scale bar, 50 μm). (H) Serum levels of cfDNA measured by ELISA. (I) qRT-PCR analysis of pancreatic tissue expression of pro-inflammatory cytokines (*Il6, Tnfa, Il1β*), neutrophil chemotaxis and activation markers (*Cxcl1, Cxcl2, S100a8, S100a9*), and genes associated with NETs formation (*Padi4, Nfkb, Nlrp3, Il17a*). Data are presented as mean ± SEM. Statistical significance was determined using an unpaired two-tailed Student’s t-test. *p<0.05, **p<0.01, ***p<0.001. Abbreviations: GF, germ-free; Cer, cerulein; AP, acute pancreatitis; MPO, myeloperoxidase; CitH3, citrullinated histone H3; cfDNA, cell-free DNA; LPS, lipopolysaccharide; NETs, neutrophil extracellular traps; qRT-PCR, real-time quantitative PCR.

Building upon prior observations of gut microbiota dysbiosis in AP patients—particularly the marked enrichment of *E. coli*—we investigated whether this bacterium plays a causative role in disease progression and NETs induction. We further performed an *in vivo* study using GF mice monocolonized with a clinically relevant enterohemorrhagic *E. coli* strain (ATCC 43895) and subsequently subjected to cerulein-induced AP (Figure 7A). Compared to GF controls, GF-*E. coli*-colonized mice exhibited significantly exacerbated pancreatic injury characterized by intensified inflammatory cell infiltration, increased acinar cell apoptosis, and elevated histopathological scores (Figure 7B–C). Consistently, serum levels of pancreatic enzymes (amylase and lipase), pro-inflammatory cytokines (TNF-α, IL-6 and IL1β) and LPS were all significantly increased in *E. coli*-colonized mice compared to GF controls (Figure 7D–E). Flow cytometric analysis further revealed a marked expansion of CD11b⁺Ly6G⁺ neutrophil populations in both spleen and bone marrow, indicative of enhanced systemic neutrophil mobilization (Figure 7F). Concurrently, pancreatic NETs formation—as assessed by MPO/CitH3 co-localization in pancreatic tissue—and serum circulating cfDNA levels were markedly elevated in the *E. coli*-colonized group (Figure 7G–H). At the transcriptional level, qRT-PCR analysis of pancreatic tissue revealed significant upregulation of genes involved in inflammatory signaling (*Il6, Tnfa* and *Il1b*), neutrophil chemotaxis and activation (*Cxcl1*, *Cxcl2, S100a8, S100a9*), and NETs- associated pathways (*Padi4, Nlrp3, Nfkb, Il17a*) (Figure 7I). Collectively, these findings provide direct evidence that *E. coli* acts as a key pathogenic driver in AP, aggravating pancreatic damage through the induction of aberrant NETs formation and amplifying both local and systemic inflammatory responses.

### Gut microbiota dysbiosis orchestrates neutrophil heterogeneity to drive excessive NETs formation in AP

**Figure 8.**
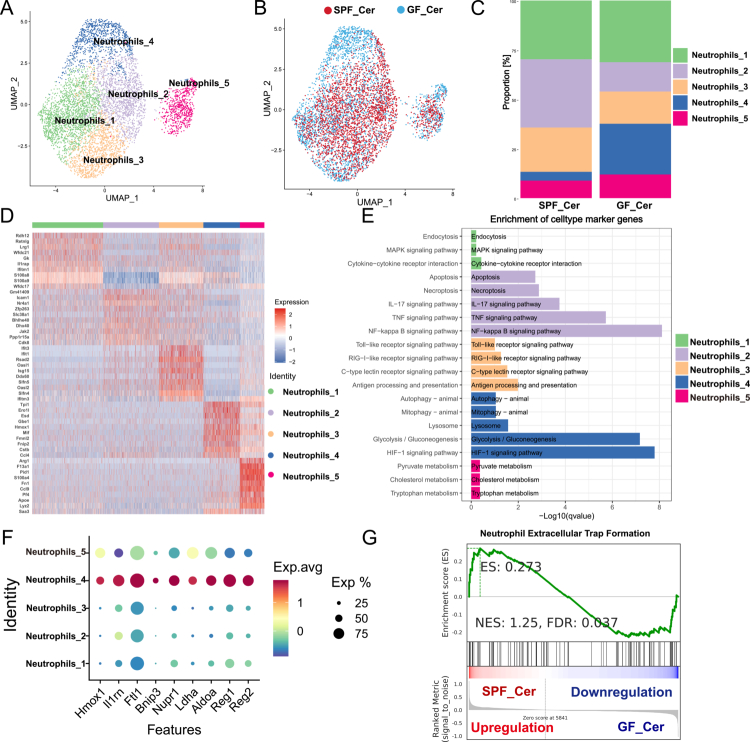
Single-cell transcriptomic analysis reveals gut microbiota dysbiosis promotes NETs formation via neutrophil Subset Reprogramming. (A) UMAP visualization of pancreatic single-cell RNA sequencing data identifies five transcriptionally distinct neutrophil clusters (Neutrophils_1 to Neutrophils_5). (B) UMAP plot illustrating differences in the distribution of neutrophil subsets between SPF_Cer and GF_Cer mice, demonstrating microbiota-dependent alterations in neutrophil heterogeneity. (C) Bar plot quantifying the relative abundance of neutrophil subpopulations across SPF_Cer and GF_Cer groups, highlighting the expansion of specific subsets in dysbiotic conditions. (D) Heatmap illustrating the expression profiles of representative genes across neutrophil subsets, delineating their functional identities. (E) KEGG pathway enrichment analysis of neutrophil clusters reveals distinct molecular signatures: Neutrophils_2 enriched in pro-inflammatory pathways including TNF, NF-κB, IL-17 signaling, apoptosis, and necroptosis; Neutrophils_3 enriched in antigen processing and Toll-like receptor signaling; Neutrophils_4 characterized by enrichment in HIF-1 signaling, glycolysis, lysosome activity, and autophagy- associated metabolic pathways. (F) Dot plot profiling key genes associated with anti-inflammatory regulation (*Hmox1* and *Il1rn*), mitochondrial protection (*Bnip3* and *Nupr1*), metabolic reprogramming (*Ldha* and *Aldoa*), and tissue repair (*Reg1* and *Reg2*) across neutrophil subpopulations. (G) GSEA focused on the Neutrophils_4 demonstrates significant enrichment of NETs formation pathways in SPF_Cer mice compared to GF_Cer mice, suggesting involvement of this subset in NETs regulation under dysbiotic conditions. Abbreviations: UMAP, uniform manifold approximation and projection; GSEA, gene set enrichment analysis; NETs, neutrophil extracellular traps; SPF, specific pathogen-free; GF, germ-free; KEGG, Kyoto Encyclopedia of Genes and Genomes.

To delineate mechanisms underlying gut microbiota dysbiosis-driven NETs hyperactivation, we performed unsupervised dimensionality reduction analysis on pancreatic neutrophils to resolve heterogeneity and phenotypic divergence within neutrophil subpopulations. This approach identified five transcriptionally and functionally distinct neutrophil subsets (Neutrophils_1–5) (Figure 8A). Notably, the distribution of these subsets differed substantially between groups: SPF mice exhibited a predominance of pro-inflammatory subsets (Neutrophils_2, Neutrophils_3, and Neutrophils_5), whereas the metabolically adaptive and potentially reparative subset Neutrophils_4 was enriched in GF mice (Figure 8B–C).

Analysis of gene expression across neutrophil subpopulations identified a unique molecular characteristics for each cluster (Figure 8D, **Table S5**). Neutrophils_2 displayed a classic "cytokine storm" phenotype, with significant enrichment of genes involved in inflammatory pathways, including NF-κB, IL-17, and TNF signaling, as well as apoptotic and necroptotic cascades (Figure 8E). Neutrophils_3 exhibited pronounced antigen-presenting features, characterized by upregulation of genes associated with Toll-like receptor (TLR), RIG-I-like receptor, and C-type lectin receptor signaling pathways, suggesting a pivotal role in early inflammation amplification during AP (Figure 8E).

In contrast, the Neutrophils_4 subset demonstrated a transcriptional program associated with immunometabolic resilience and tissue protection(Figure 8E). In agreement with the pathway analysis, Neutrophils_4 displayed elevated expression of anti-inflammation (*Il1rn*), antioxidant defense (*Hmox1, Ftl1*), regulators of mitochondrial integrity and stress adaptation (*Bnip3, Nupr1*), glycolytic enzymes (*Ldha, Aldoa*), and tissue repair factors (*Reg1, Reg2*) (Figure 8F). These transcriptional features were validated at the tissue level by qRT-PCR and S100A8/HMOX-1 co‑staining **(Figure S5)**. However, GSEA revealed that Neutrophils_4 from SPF mice were also enriched for NETosis-associated pathways (Figure 8G), suggesting that under conditions of gut microbiota dysbiosis, even functionally protective subsets may be co-opted towards pathogenic NETs formation. In conclusion, these findings suggest that gut microbiota dysbiosis not only alters the proportional composition of pancreatic neutrophil subsets but also reprograms their functional states, promoting a pro-NETotic landscape that contributes to exacerbated inflammation and tissue damage in AP.

## Discussion

Although gut microbiota dysbiosis has long been implicated in the pathogenesis and the severity of AP, the specific bacterial species contributing to disease progression and their precise immunological mechanisms remain inadequately defined. In this study, we provide compelling evidence that intestinal microbiota dysbiosis, particularly characterized by an abnormal expansion of *Escherichia coli*, profoundly alters the host immune landscape. Mechanistically, this dysbiosis triggers emergency granulopoiesis within the bone marrow and activates the TLR2/NLRP3 signaling axis, leading to the expansion of pro-inflammatory neutrophil subsets (Neutrophils_2 and Neutrophils_3) while depleting anti-inflammatory neutrophils (Neutrophils_4) with putative NETs-suppressive and tissue-reparative capacities. The resulting immunological skew promotes aberrant NETs formation, which significantly amplifies pancreatic and systemic inflammation, thereby accelerating AP progression.

Previous studies have shown that GF conditions impair innate immune cell development, most notably neutrophils, monocytes, macrophages, and splenic CD4⁺ T cells, highlighting the indispensable role of the microbiota in maintaining immune homeostasis^28‐30^. Consistent with these observations, our data demonstrate that GF mice exhibit significantly attenuated AP, characterized by reduced emergency granulopoiesis, diminished neutrophil activation and NETosis, and decreased systemic inflammation. Dysbiotic microbiota markedly amplified these immune responses, exacerbating severity of AP. In contrast, microbiota reconstitution using fecal material from AP patients markedly restored and exacerbated neutrophil-driven inflammatory responses in GF mice, including widespread expansion and mobilization of neutrophils across the bone marrow, spleen, circulation, and pancreas. This findings corroborate the model proposed by Clarke et al., wherein translocated gut-derived microbial-associated molecular patterns (MAMPs) modulate bone marrow hematopoiesis by influencing the activity of granulocyte-monocyte progenitors (GMPs)^31^. Moreover, microbiota dysbiosis significantly reshapes the splenic immune architecture, as evidenced by increased frequencies of CD11b⁺ myeloid cells and CD3⁺/CD4⁺ T cells—an immunological signature resembling those observed in gut-derived sepsis and critical illness^32‐34^. Importantly, this study provides the first integrated evidence of global neutrophil system activation driven by microbial imbalance across multiple immune-relevant tissues, advancing our understanding beyond prior organ-specific investigations.^19^,^35^,^36^

Recent studies have revealed that neutrophils are not a homogeneous population, but comprise functionally distinct subsets shaped by the microenvironment.^34^,^37^,^38^ Using single-cell transcriptomics, Zhang et al. previously identified two distinct neutrophil subsets—pro-inflammatory (N1, *Tnf* ^*+*^) and anti-inflammatory (N2, *Arg1*^*+*^)—within the pancreas and peripheral blood of AP models, demonstrating differential localization and pathological roles.^39^ The pro-inflammatory subset was predominantly localized in inflammatory regions, significantly promoting tissue damage and NETs formation, while the anti-inflammatory subset appeared to facilitate tissue repair and fibrosis. Building upon this concept, our study highlights that gut microbiota composition fundamentally reprograms neutrophil heterogeneity in AP. Specifically, we observed marked enrichment of pro-inflammatory neutrophil subsets (Neutrophils_2 and Neutrophils_3) in SPF mice, characterized by activation of NETosis-associated signaling cascades including NF-κB and IL-17, whereas, the metabolically protective Neutrophils_4 subset was predominant in GF conditions. While both studies underscore the importance of neutrophil subset heterogeneity, our study emphasizes the upstream role of microbial cues in modulating these populations through emergency granulopoiesis and NETotic priming, suggesting a context-specific regulatory mechanism influenced by microbial composition, tissue localization, and disease stage.

The pathological role of NETs in AP has been well established, contributing to acinar cell necrosis, microvascular thrombosis, and multi-organ dysfunction. Pharmacological inhibition of NETs formation has consistently attenuated disease severity in both preclinical animal models and *ex-vivo* human studies.^16^,^18^,^40^,^41^ Expanding upon this, our data demonstrate that gut microbiota dysbiosis—driven primarily by *Escherichia coli* enrichment—acts as a potent inducer of NETs formation, thereby exacerbating pancreatic injury and systemic inflammation. Specifically, SPF-Cer mice exhibited significant upregulation of canonical NETosis mediators (e.g., *Padi4* and *Nlrp3*) in pancreatic tissues, and bone marrow-derived neutrophils from these animals generated heightened NETs responses upon stimulation. Clinically, serum levels of cfDNA, a biomarker for NETs, were elevated in AP patients and positively correlated with intestinal *E. coli* abundance. These findings align with previous reports in hypertriglyceridemia-induced pancreatitis and abdominal aortic aneurysm, where microbiota-derived NETosis signals were similarly implicated in exacerbating disease severity^19^,^35^, suggesting NETs formation a universal response to microbiota imbalance. Nonetheless, the effect of microbiota on NETs formation appears to be disease specific. For instance, Ascher et al. reported increased NETosis in GF models of mesenteric ischemia-reperfusion (I/R) due to loss of microbiota-induced endotoxin tolerance,^42^ in contrast to our findings of reduced NETosis in GF mice. These conflicting results underscore the nuanced, context-dependent complexity of microbiota-host immune interactions.

The innovative value of this study lies in clearly identifying gut-derived *Escherichia coli* as a core proinflammatory pathogen in aggravating AP, through a multilayered screening strategy. However, multiple potential mechanisms might be involved in the *E. coli*-driven formation of NETs and exacerbation of pancreatic injury. First, LPS derived from *E. coli* can activate NF-κB and oxidative stress signaling via the TLR4/MyD88 pathway, subsequently inducing PAD4-dependent chromatin decondensation and directly promoting NETs formation. Additionally, LPS can also trigger immunothrombosis via platelet-expressed TLR4, further amplifying NETosis.^43^,^44^ This mechanism aligns well with our observations of elevated expression of NETs-related genes (such as *Padi4, Nlrp3, Nfkb, Il17a*) in pancreatic tissues, as well as significantly increased serum cfDNA levels in our experimental models. Recently, Zheng et al. demonstrated that commensal *E. coli* (MG1655) aggravates intestinal barrier disruption and systemic inflammation through activation of the TLR4/MyD88/p38-MAPK pathway and endoplasmic reticulum stress (ERS), further supporting the critical role of *E. coli* in activating TLR4 signaling pathways.^45^

Moreover, other virulence factors from *E. coli* may act synergistically to enhance NETosis. For instance, α-hemolysin (HlyA) from uropathogenic *E. coli (UPEC)* promotes NETosis by activating the NLRP3 inflammasome^46^; type I fimbrial adhesin (FimH) directly binds to TLR4 and synergistically enhances inflammatory signaling^47^; and outer membrane vesicles (OMVs) effectively deliver LPS and induce IL-8/CXCL1 release, significantly enhancing neutrophil recruitment and NETs formation^48^,^49^. It is noteworthy that diverse strains may display differential regulation of NETosis—for example, TcpC protein can negatively regulate NETosis by promoting PAD4 degradation^50^,^51^. Furthermore, strain-specific genetic variations, such as structural variants involving carbohydrate metabolism genes (e.g., scrK deficiency), significantly amplify mucosal inflammation and barrier disruption under high-fructose dietary conditions^52^. Certain bacterial metabolites, such as indoxyl sulfate (IS), might indirectly regulate NETosis via activation of aryl hydrocarbon receptor (AHR) signaling and induce distant organ injury, although the direct mechanistic links with AP require further elucidation in future studies.^53^

Nevertheless, this study has several limitations. First, FMT donors were restricted to SAP, preventing direct comparison with mild disease; Second, the molecular mechanisms by which *E. coli* promotes NETosis, including the LPS-TLR2 axis, the NLRP3 inflammasome, and potential virulence factors, remain to be defined, studies using pathway inhibitors and mutant strains are needed; Third, functional validation of the protective Neutrophils_4 subset is currently restricted to transcriptomic signatures. Additional *in vitro* and *in vivo* assays are needed to define their role in NETs formation and tissue repair; Lastly, while our clinical correlations are compelling, larger patient cohorts are required to robustly validate the association between *E. coli* abundance and cfDNA levels.

In summary, this study provides a comprehensive framework linking gut microbiota dysbiosis, particularly *E. coli* overgrowth, to immune dysregulation, neutrophil subset remodeling, and enhanced NETosis in AP. For the first time, we identify *E. coli* as a critical upstream effector of AP severity and propose that microbiota-directed therapies and NETosis pathway inhibition may represent promising strategies for clinical intervention.

## Supplementary Material

Supplementary Material Table S5.xlsx

Supplementary Material Table S1. General characteristics.Table S2. Demographic and clinical characteristics of FMT donors.Table S3. Primer sequences used for qPCR assay.

Supplementary Material Figure S1. Flow cytometry gating strategy for immune cell identification. (A) Representative gating scheme for delineating immune cell populations from splenic tissue. (B) Gating schema applied to immune cells isolated from peripheral blood and bone marrow.Figure S2. Transcriptional landscape of the pancreatic immune microenvironment in response to gut microbiota dysbiosis. (A) UMAP visualization illustrating 16 distinct cell clusters derived from integrated single-cell RNA sequencing datasets of pancreatic tissues from SPF_Cer and GF_Cer mice. (B) Dot plot displaying canonical marker genes expression profiles across key cell types, including macrophages, monocytes, neutrophils, fibroblasts, acinar cells, and T cells. (C) KEGG pathway enrichment analysis of genes upregulated in SPF_Cer mice reveals robust activation of inflammatory and immune signaling cascades, notably the TNF, IL-17, and NF-κB pathways. (D) Circos plot mapping significantly upregulated genes in SPF_Cer mice to enriched KEGG pathways, highlighting functional interactions and pathway convergence. (E) GO enrichment analysis of genes upregulated in SPF_Cer indicates prominent enrichment of terms related to immune response, chemotaxis, and transcriptional regulation. (F) Circos plot displaying the distribution of differentially expressed genes across the top enriched GO biological processes.Figure S3. Gut microbiota dysbiosis promotes activation of inflammatory pathways and suppresses acinar repair gene Reg3b in the pancreas. (A) Heatmap illustrating differentially expressed genes in pancreatic tissues of SPF_Cer versus GF_Cer mice. Genes involved in antimicrobial defense and epithelial repair (e.g., Reg3b) were predominantly upregulated in GF_Cer mice, whereas inflammasome-related genes were more abundant in SPF_Cer samples. (B) Western blot analysis of TLR2, NLRP3, ASC, and Reg3β protein levels in pancreatic lysates reveals increased expression of NLRP3 inflammasome components in SPF_Cer mice. (C) Violin plot depicting specific expression of *Reg3b*in acinar cells. (D) UMAP projection showing elevated *Reg3b* expression in the pancreas of GF_Cer mice compared to SPF_Cer controls. (E) Immunofluorescence staining confirms elevated Reg3b protein co-localizing with amylase in acinar cells under GF conditions. (F) Violin plot illustrating *Nlrp3* expression enriched in macrophages, monocytes, and neutrophils. (G) UMAP plot demonstrating increased *Nlrp3* expression in SPF_Cer compared to GF_Cer pancreatic tissues. (H) Representative immunofluorescence images revealing increased co-localization of Ly6G and NLRP3 in SPF_Cer mice, indicating enhanced inflammasome activation within pancreatic neutrophils under dysbiotic conditions.Figure S4. (A) qRT-PCR validation of NETosis-associated genes identified by scRNA-seq. (B) Relative abundances of fecal *E. coli* and *K. pneumoniae* following antibiotic treatments, measured by qPCR.Figure S5. Validation of Neutrophil subset 4 (Neutrophils_4) markers at the protein and transcript levels in murine acute pancreatitis.

Supplementary Material Table S4.xlsx

## Data Availability

The data (fecal metagenomic sequencing data from AP patients) presented in the study have been deposited in the Sequence Read Archive (SRA) under accession number PRJNA1135945 (https://www.ncbi.nlm.nih.gov/sra). The raw single-cell sequencing data of mouse pancreas from this study have been deposited in the Gene Expression Omnibus (GEO) under accession number GSE301305 (https://www.ncbi.nlm.nih.gov/geo/query/acc.cgi).

## Abbreviations

AP: acute pancreatitis
ASC: apoptosis-associated speck-like protein containing a CARD
BSA: bovine serum albumin
cfDNA: cell-free DNA
DC: dendritic cell
*E. coli*: Escherichia coli
ECL: enhanced chemiluminescence
ERS: endoplasmic reticulum stress
FMT: fecal microbiota transplantation
GF: germ-free
GMP: granulocyte-monocyte progenitor
GO: Gene Ontology
GSEA: Gene Set Enrichment Analysis
G^−^: Gram-negative
G^+^: Gram-positive
H&E: hematoxylin and eosin
HRP: horseradish peroxidase
IL: interleukin
KEGG: Kyoto Encyclopedia of Genes and Genomes
MAPK: mitogen-activated protein kinase
MAMP: microbe-associated molecular pattern
MODS: multiple organ dysfunction syndrome
MOF: multiple organ failure
MyD88: myeloid differentiation primary response 88; Neo, neomycin
NETs: neutrophil extracellular traps
NEO: neomycin
NLRP3: nucleotide-binding oligomerization domain-like receptor family pyrin domain containing 3
PCA: principal component analysis
PBS: phosphate-buffered saline
PMA: phorbol 12-myristate 13-acetate
qRT-PCR: quantitative real-time polymerase chain reaction
SAP: severe acute pancreatitis
SCFA: short-chain fatty acid
scRNA-seq: single-cell RNA sequencing
SIRS: systemic inflammatory response syndrome
SPF: specific pathogen-free
Treg: regulatory T cell
Th17: T-helper 17 cell
TLR: Toll-like receptor
TLR2: Toll-like receptor 2
TLR4: Toll-like receptor 4
TNF-α: tumor necrosis factor-α
UMAP: Uniform Manifold Approximation and Projection
UMI: unique molecular identifier
VAN: vancomycin
