# Supplementary material for: Gut microbiota dysbiosis exacerbates acute pancreatitis via Escherichia coli-driven neutrophil heterogeneity and NETosis
Source: Gut Microbes. 2025 Dec 24;18(1):2606480. doi: 10.1080/19490976.2025.2606480 (PMC12758309; doi:10.1080/19490976.2025.2606480)
Supplement: Supplementary Material — Table S1. General characteristics.Table S2. Demographic and clinical characteristics of FMT donors.Table S3. Primer sequences used for qPCR assay. [file KGMI_A_2606480_SM6796.docx]

**Table S1.** General characteristics

| **Variables** | Total  (n = 52) | AP  (n = 26) | HC  (n = 26) | *p* |
| --- | --- | --- | --- | --- |
| Male, n (%) | 38 (73.1) | 20 (76.9) | 18 (69.2) | 0.532 |
| Age (years) | 46.1 ± 12.4 | 44.5 ± 10.2 | 48.2 ± 14.9 | 0.335 |
| BMI | 26.0 ± 3.6 | 26.7 ± 3.2 | 24.9 ± 3.9 | 0.13 |
| Smoking, n (%) | 16 (30.8) | 7 (26.9) | 9 (34.6) | 0.548 |
| Drinking, n (%) | 24 (46.2) | 14 (53.8) | 10 (38.5) | 0.266 |
| Hypertensive disease, n (%) | 38 (73.1) | 20 (76.9) | 18 (69.2) | 0.532 |
| Diabetes, n (%) | 46.1 ± 12.4 | 44.5 ± 10.2 | 48.2 ± 14.9 | 0.335 |

**Abbreviations:** AP, acute pancreatitis; HC, healthy control

**Table S2.** Primer sequences used for qPCR assay

| Gene | Species | Primers (5'-3') |
| --- | --- | --- |
| *Gapdh* | Mouse | Forward: AGGTCGGTGTGAACGGATTTG  Reverse: GGGGTCGTTGATGGCAACA |
| *IL1b* | Mouse | Forward: GAAATGCCACCTTTTGACAGTG  Reverse: TGGATGCTCTCATCAGGACAG |
| *IL6* | Mouse | Forward: CTGCAAGAGACTTCCATCCAG  Reverse: AGTGGTATAGACAGGTCTGTTGG |
| *Tnfa* | Mouse | Forward: CAGGCGGTGCCTATGTCTC  Reverse: CGATCACCCCGAAGTTCAGTAG |
| *Nlrp3* | Mouse | Forward: ATTACCCGCCCGAGAAAGG  Reverse: CATGAGTGTGGCTAGATCCAAG |
| *Il17a* | Mouse | Forward: CAGACTACCTCAACCGTTCCAC  Reverse: TCCAGCTTTCCCTCCGCATTGA |
| *Nfkb* | Mouse | Forward: TCCTGTTCGAGTCTCCATGCAG  Reverse: GGTCTCATAGGTCCTTTTGCGC |
| *Padi4* | Mouse | Forward: TGGTCCTCCAGTCAAGAAGAG  Reverse: GCTTTCACCTGTAGGGTCACC |
| *Cxcl1* | Mouse | Forward: ACTGCACCCAAACCGAAGTC  Reverse: TGGGGACACCTTTTAGCATCTT |
| *Cxcl2* | Mouse | Forward: CCAACCACCAGGCTACAGG  Reverse: GCGTCACACTCAAGCTCTG |
| *S100a8* | Mouse | Forward: AAATCACCATGCCCTCTACAAG  Reverse: CCCACTTTTATCACCATCGCAA |
| *S100a9* | Mouse | Forward: GCACAGTTGGCAACCTTTATG  Reverse: TGATTGTCCTGGTTTGTGTCC |
| *Tlr2* | Mouse | Forward: CTCTTCAGCAAACGCTGTTCT  Reverse: GGCGTCTCCCTCTATTGTATTG |
| *Nfkb1* | Mouse | Forward: ATGGCAGACGATGATCCCTAC  Reverse: CGGAATCGAAATCCCCTCTGTT |
| *Fpr2* | Mouse | Forward: CCGTCCTTTACGAGTCCTTACA  Reverse: CAGGAGGTGAAGTAGAACTGGT |
| *Itgal* | Mouse | Forward: CCAGACTTTTGCTACTGGGAC  Reverse: GCTTGTTCGGCAGTGATAGAG |
| *Casp4* | Mouse | Forward: AGCGTTGGGTTTTTGTAGATGC  Reverse: CCTTGTGAACTCTTCAGGGGA |
| *Syk* | Mouse | Forward: CTACCTGCTACGCCAGAGC  Reverse: TTCCCTCTCGATGGTGTAGTG |
| *Plcb1* | Mouse | Forward: AGACCTGGTGAACATTTCCCA  Reverse: ACAAGCCTCTAGTGCAGTTTC |
| *Hmox1* | Mouse | Forward: AGGTACACATCCAAGCCGAGA  Reverse: CATCACCAGCTTAAAGCCTTCT |
| *Il1rn* | Mouse | Forward: TAGACATGGTGCCTATTGACCT  Reverse: TCGTGACTATAAGGGGCTCTTC |
| *Ftl1* | Mouse | Forward: CGTCAGAATTATTCCACCGAGG  Reverse: GCCACGTCATCCCGATCAAA |
| *Bnip3* | Mouse | Forward: CTGGGTAGAACTGCACTTCAG  Reverse: GGAGCTACTTCGTCCAGATTCAT |
| *Nupr1* | Mouse | Forward: ACCCTTCCCAGCAACCTCTAA  Reverse: TCTTGGTCCGACCTTTCCGA |
| *Ldha* | Mouse | Forward: CAAAGACTACTGTGTAACTGCGA  Reverse: TGGACTGTACTTGACAATGTTGG |
| *Aldoa* | Mouse | Forward: AGTCCACCGGAAGCATTGC  Reverse: CAGCCCCTGGGTAGTTGTC |
| *Reg1* | Mouse | Forward: CTCATGCCTGATCGTCCTGTC  Reverse: AGCCCAAGTTAAACGGTCTTC |
| *Reg2* | Mouse | Forward: CCTGATGTTCCTGTCATACAGC  Reverse: CCAGGTCAAACGGTCTTCAAT |
| *E.coil* | Bacteria | Forward: CATGCCGCGTGTATGAAGAA  Reverse: CGGGTAACGTCAATGAGCAAA |
| *K.p* | Bacteria | Forward: ACGCTGTCACGACGCTATC  Reverse: GGCTGAGGCACGGTATCC |
| *Eubacteria* | Bacteria | Forward: ACTCCTACGGGAGGCAGCAG  Reverse: ATTACCGCGGCTGCTGG |

**Table S3.** Demographic and clinical characteristics of FMT donors

| **Variables** | Total  (n = 10) | AP  (n = 5) | HC  (n = 5) | *p* |
| --- | --- | --- | --- | --- |
| Male, n (%) | 6 (60.0) | 3 (60) | 3 (60) | 1 |
| Age (years) | 46.6 ± 9.1 | 48.2 ± 12.0 | 45.0 ± 5.8 | 0.607 |
| Smoking, n (%) | 3 (30.0) | 2 (40) | 1 (20) | 1 |
| Drinking, n (%) | 5 (50.0) | 2 (40) | 3 (60) | 1 |
| Hypertensive disease, n (%) | 3 (30.0) | 1 (20) | 2 (40) | 1 |
| Diabetes, n (%) | 1 (10.0) | 1 (20) | 0 (0) | 1 |

**Abbreviations:** AP, acute pancreatitis; HC, healthy control
