# Supplementary material for: Gut microbiota dysbiosis exacerbates acute pancreatitis via Escherichia coli-driven neutrophil heterogeneity and NETosis
Source: Gut Microbes. 2025 Dec 24;18(1):2606480. doi: 10.1080/19490976.2025.2606480 (PMC12758309; doi:10.1080/19490976.2025.2606480)
Supplement: Supplementary Material — Figure S1. Flow cytometry gating strategy for immune cell identification. (A) Representative gating scheme for delineating immune cell populations from splenic tissue. (B) Gating schema applied to immune cells isolated from peripheral blood and bone marrow.Figure S2. Transcriptional landscape of the pancreatic immune microenvironment in response to gut microbiota dysbiosis. (A) UMAP visualization illustrating 16 distinct cell clusters derived from integrated single-cell RNA sequencing datasets of pancreatic tissues from SPF_Cer and GF_Cer mice. (B) Dot plot displaying canonical marker genes expression profiles across key cell types, including macrophages, monocytes, neutrophils, fibroblasts, acinar cells, and T cells. (C) KEGG pathway enrichment analysis of genes upregulated in SPF_Cer mice reveals robust activation of inflammatory and immune signaling cascades, notably the TNF, IL-17, and NF-κB pathways. (D) Circos plot mapping significantly upregulated genes in SPF_Cer mice to enriched KEGG pathways, highlighting functional interactions and pathway convergence. (E) GO enrichment analysis of genes upregulated in SPF_Cer indicates prominent enrichment of terms related to immune response, chemotaxis, and transcriptional regulation. (F) Circos plot displaying the distribution of differentially expressed genes across the top enriched GO biological processes.Figure S3. Gut microbiota dysbiosis promotes activation of inflammatory pathways and suppresses acinar repair gene Reg3b in the pancreas. (A) Heatmap illustrating differentially expressed genes in pancreatic tissues of SPF_Cer versus GF_Cer mice. Genes involved in antimicrobial defense and epithelial repair (e.g., Reg3b) were predominantly upregulated in GF_Cer mice, whereas inflammasome-related genes were more abundant in SPF_Cer samples. (B) Western blot analysis of TLR2, NLRP3, ASC, and Reg3β protein levels in pancreatic lysates reveals increased expression of NLRP3 inflammasome components in SPF_Cer mice. (C) Vi [file KGMI_A_2606480_SM6795.docx]

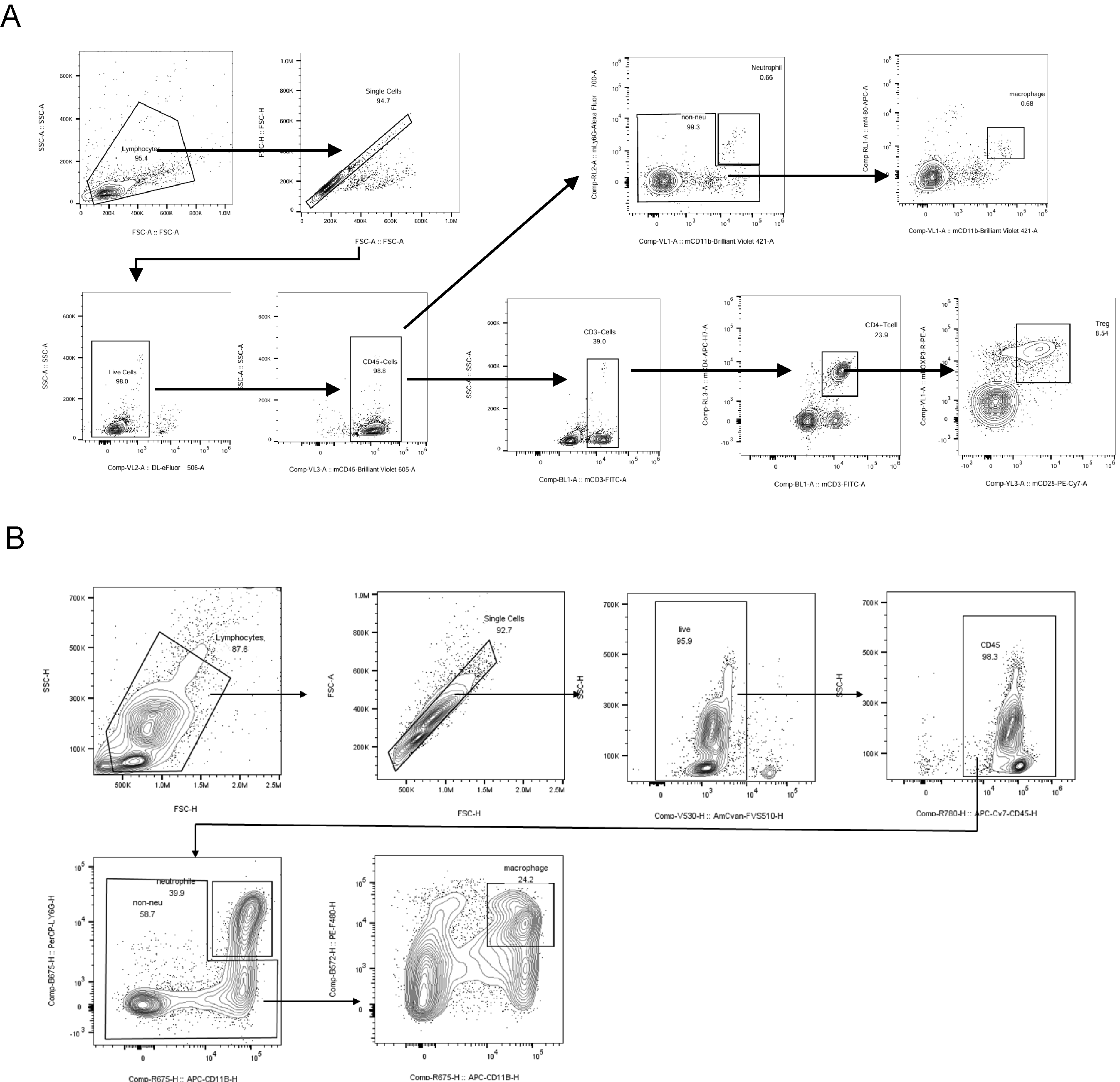
**Figure S1. Flow cytometry gating strategy for immune cell identification.** (A) Representative gating scheme for delineating immune cell populations from splenic tissue. (B) Gating schema applied to immune cells isolated from peripheral blood and bone marrow.


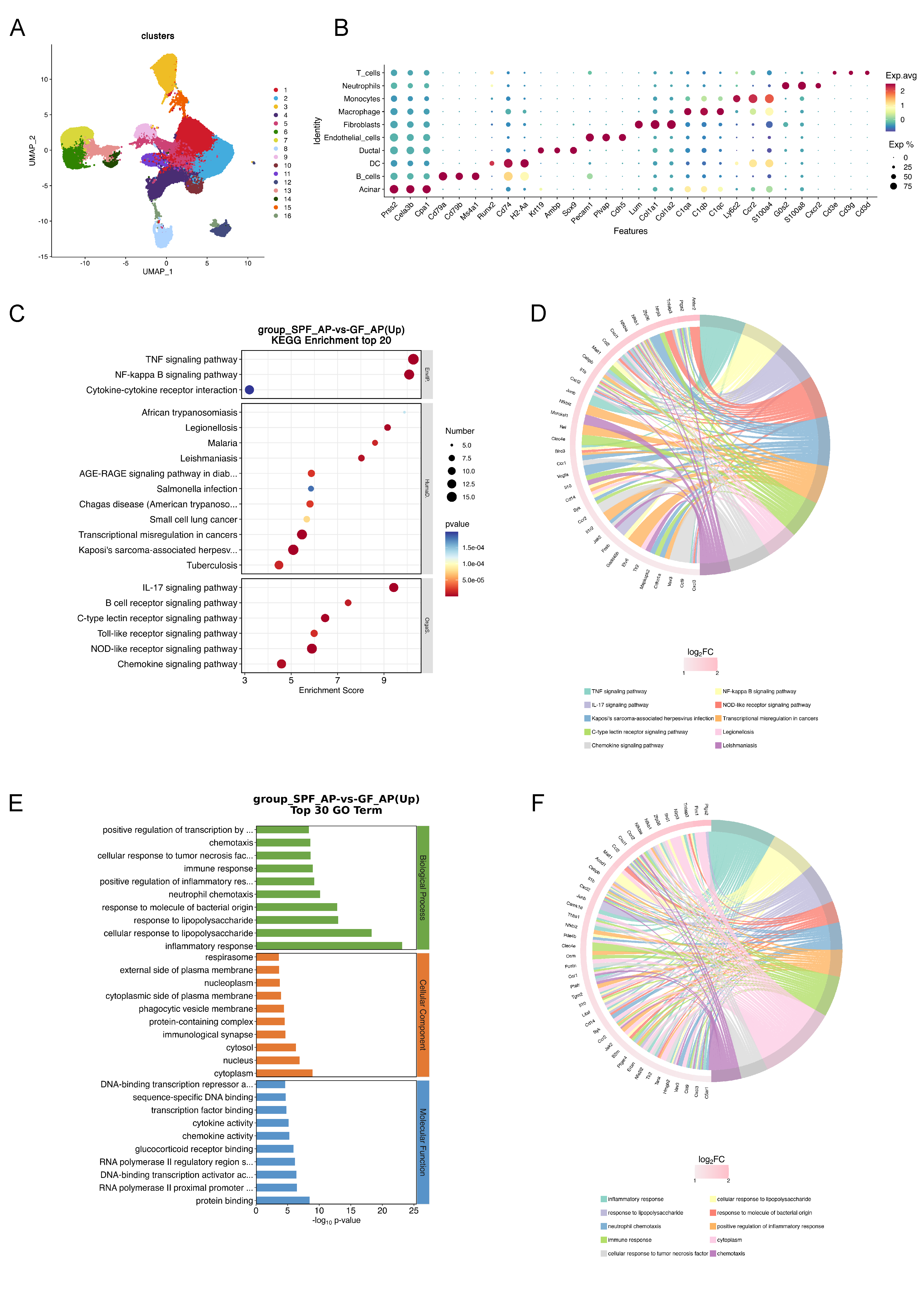


**Figure S2. Transcriptional landscape of the pancreatic immune microenvironment in response to gut microbiota dysbiosis.** (A) UMAP visualization illustrating 16 distinct cell clusters derived from integrated single-cell RNA sequencing datasets of pancreatic tissues from SPF_Cer and GF_Cer mice. (B) Dot plot displaying canonical marker genes expression profiles across key cell types, including macrophages, monocytes, neutrophils, fibroblasts, acinar cells, and T cells. (C) KEGG pathway enrichment analysis of genes upregulated in SPF_Cer mice reveals robust activation of inflammatory and immune signaling cascades, notably the TNF, IL-17, and NF-κB pathways. (D) Circos plot mapping significantly upregulated genes in SPF_Cer mice to enriched KEGG pathways, highlighting functional interactions and pathway convergence. (E) GO enrichment analysis of v upregulated in SPF_AP indicates prominent enrichment of terms related to immune response, chemotaxis, and transcriptional regulation. (F) Circos plot displaying the distribution of differentially expressed genes across the top enriched GO biological processes.


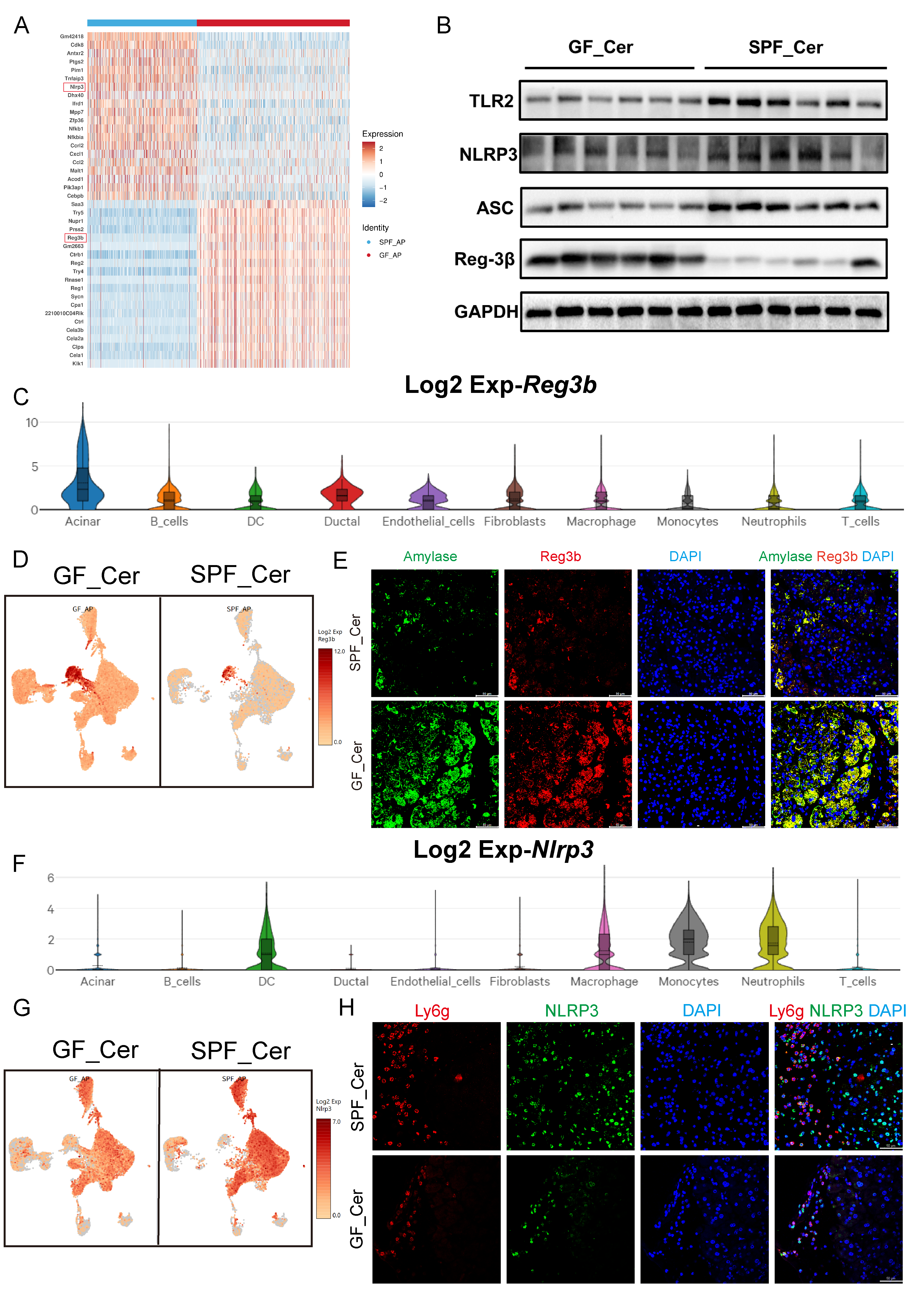
 **Figure S3. Gut microbiota dysbiosis promotes activation of inflammatory pathways and suppresses acinar repair gene Reg3b in the pancreas.** (A) Heatmap illustrating differentially expressed genes in pancreatic tissues of SPF_Cer versus GF_Cer mice. Genes involved in antimicrobial defense and epithelial repair (e.g., Reg3b) were predominantly upregulated in GF_Cer mice, whereas inflammasome-related genes were more abundant in SPF_Cer samples. (B) Western blot analysis of TLR2, NLRP3, ASC, and Reg3β protein levels in pancreatic lysates reveals increased expression of NLRP3 inflammasome components in SPF_Cer mice. (C) Violin plot depicting specific expression of *Reg3b* in acinar cells. (D) UMAP projection showing elevated *Reg3b* expression in the pancreas of GF_Cer mice compared to SPF_Cer controls. (E) Immunofluorescence staining confirms elevated Reg3b protein co-localizing with amylase in acinar cells under GF conditions. (F) Violin plot illustrating *Nlrp3* expression enriched in macrophages, monocytes, and neutrophils. (G) UMAP plot demonstrating increased Nlrp3 expression in SPF_Cer compared to GF_Cer pancreatic tissues. (H) Representative immunofluorescence images revealing increased co-localization of Ly6G and NLRP3 in SPF_Cer mice, indicating enhanced inflammasome activation within pancreatic neutrophils under dysbiotic conditions.


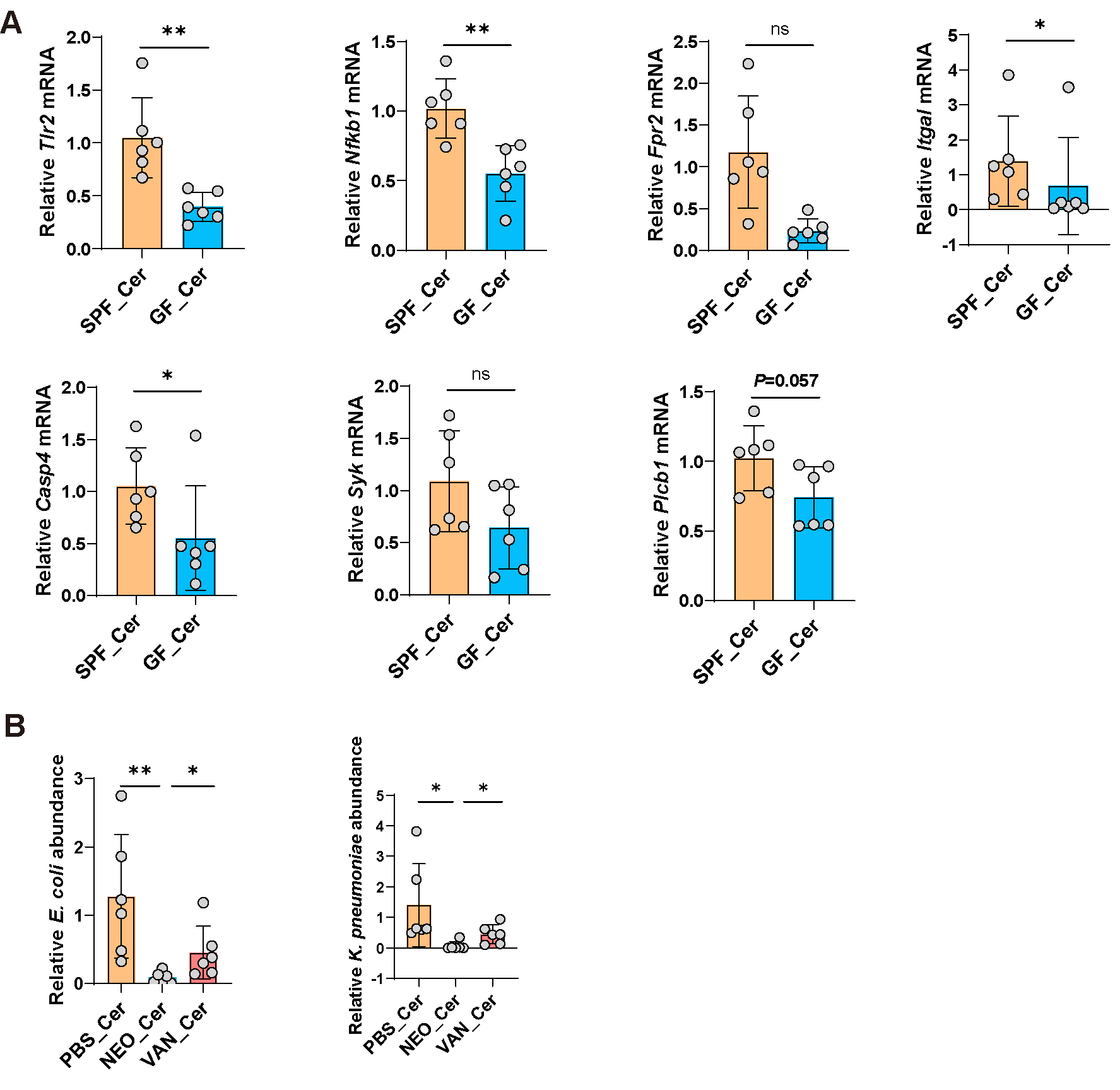


**Figure S4. (A)qRT-PCR validation of NETosis-associated genes identified by scRNA-seq. (B)** **Relative abundances of fecal E. coli and K. pneumoniae following antibiotic treatments, measured by qPCR.**


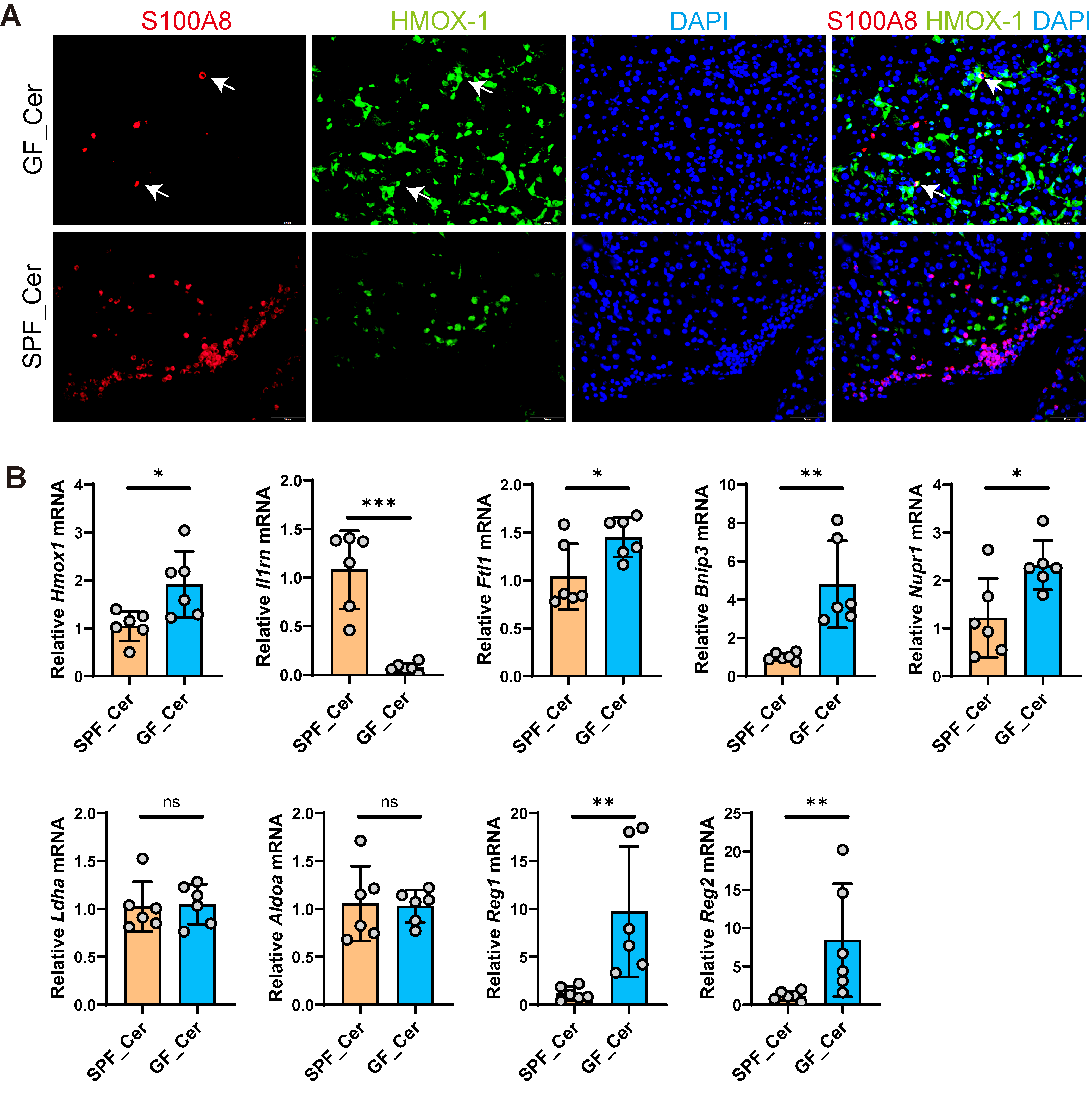


**Figure S5. Validation of Neutrophil subset 4 (Neutrophils_4) markers at the protein and transcript levels in murine acute pancreatitis.**
